# TROAP Promotes Breast Cancer Proliferation and Metastasis

**DOI:** 10.1155/2019/6140951

**Published:** 2019-05-06

**Authors:** Kai Li, Ruo Zhang, Minjie Wei, Li Zhao, Yu Wang, Xinxin Feng, Yongheng Yang, Shucai Yang, Lei Zhang

**Affiliations:** ^1^Department of Oncology I, Shengjing Hospital of China Medical University, Shenyang, China; ^2^Shuwen Biotech Co. Ltd., Deqing, China; ^3^School of Pharmacy, China Medical University, Shenyang, China; ^4^The 4^th^ Affiliated Hospital of Harbin Medical University, Harbin, China; ^5^Department of Pathology, Harbin Medical University, Harbin, China; ^6^Department of Anatomy, Basic Medical Science College, Harbin Medical University, Harbin, China

## Abstract

Trophinin-associated protein (TROAP) is a cytoplasmic protein required for microtubular cytoskeleton regulation and spindle assembly, and its expression plays a critical role in the initiation and progression of various types of cancer. However, little is known about the role of TROAP in breast cancer (BC). TROAP mRNA expression levels and clinical data from Gene Expression Omnibus (GEO) datasets (GSE42568, 104 BC patients; GSE1456, 159 BC patients; and GSE21653, 266 BC patients) were analyzed by the R2: Genomics Analysis and Visualization Platform to estimate overall survival (OS). We also analyzed the genes correlated with TROAP by gene ontology (GO) enrichment analysis and Kyoto Encyclopedia of Genes and Genomes (KEGG) analysis to predict potential relationships between TROAP and other genes in BC. Our study verified that both TROAP mRNA and protein expression levels were upregulated in human BC samples and cell lines. In vitro experiments demonstrated that TROAP knockdown significantly inhibited cell proliferation, the G1 to S phase transition, and the migration and invasion abilities of BC cells. The present study suggests that TROAP plays an important role in promoting the proliferation, invasion, and metastasis of BC.

## 1. Introduction

Breast cancer (BC) is the most common cancer in women worldwide [1]. BC is a complicated heterogenous disease and the leading cause of death among the female population [2]. In 2018, it was reported that over 266,000 new cases of invasive BC were diagnosed in women in the U.S., along with nearly 64,000 new cases of noninvasive BC [3, 4], characterized by different clinical manifestations and outcomes [5]. Although previous studies have identified various biomarkers for predicting disease development and evaluating prognosis, the identification of better biomarkers and therapeutic targets is still important to improve current therapeutic strategies and outcomes for patients with BC.

Trophinin-associated protein (TROAP, also known as TASTIN), a cytoplasmic protein required for microtubular cytoskeleton regulation, was first identified as a participant in early embryo implantation [6, 7]. During cell mitosis [8], TROAP is required for mediating spindle assembly and centrosome integrity. Recently, TROAP was found to participate in the proliferation, invasion, and migration of many cancers. The upregulation of trophinin promoted the metastatic potential in human gallbladder cancer cells, which was correlated with high expression of integrin alpha3, MMP-7, MMP-9, and Ets-1 [9]. TROAP is highly expressed in ovarian cancer cell lines and positively associated with poor prognosis in ovarian cancer; thus, TROAP is regarded as a prognostic marker in ovarian cancer [10]. TROAP also enhanced the invasion of colorectal cells through a mechanism involving HMGB1/RAGE [11]. However, the involvement of TROAP in liver cancer is controversial: Jiao Y estimated the overall survival (OS) of liver cancer patients based on an analysis of The Cancer Genome Atlas Liver Hepatocellular Carcinoma (TCGA-LIHC) data and evaluated the relationship between TROAP expression in hepatocellular carcinoma (HCC) tissue and clinicopathologic parameters. Their work verified that high TROAP expression is an independent predictive marker of poor survival in liver cancer [12]. On the other hand, Lian Y reported that TROAP suppressed HCC cell growth and migration based on the study involving TROAP depletion and overexpression in HCC cell lines and tissue [13]. TROAP expression was upregulated in gastric cancer (GC) tissues compared with control tissues; thus, high TROAP expression was associated with poor survival in patients with GC. Knockdown of TROAP significantly inhibited cell proliferation, the G1 to S phase transition, and the migration and invasion abilities of GC cells [14]. These studies strongly suggest the potential that the dysregulation of TROAP expression plays a critical role in the initiation and progression of many types of cancer. However, little is known about the role of TROAP in BC.

To clarify TROAP function in BC, we analyzed TROAP expression in BC tissues from three Gene Expression Omnibus (GEO) databases and estimated OS using an R2 analysis platform. Gene correlation analysis, GO biological process analysis, and KEGG pathway analysis were performed at transcriptome level. We determined TROAP mRNA and protein levels in BC cell lines and tissues and then constructed TROAP overexpression and knockdown cell models to verify the function of TROAP in BC. The results suggest a significant role for TROAP in human BC growth and metastasis.

## 2. Materials and Methods

### 2.1. Solutions and Drugs

Mouse polyclonal anti-TROAP antibody (SAB1406913) was purchased from Sigma (Sigma-Aldrich, St. Louis, MO, USA). Mouse anti-human GAPDH antibody (sc-32233), HRP-conjugated goat anti-rabbit IgG (sc-2004), and HRP-conjugated goat anti-mouse IgG (sc-2005) were purchased from Santa Cruz Biotechnology, Inc. TRIzol reagent and an ECL Substrate Reagent Kit were purchased from Invitrogen (Grand Island, NY, USA). Western blotting (WB) equipment and materials were purchased from Sigma-Aldrich (St. Louis, MO, USA).

### 2.2. Database Analysis

Our study estimated survival with the Kaplan-Meier method based on data from three GEO datasets, evaluated TROAP mRNA levels in BC and noncancerous tissues, and analyzed the different molecular subtypes of BC in TCGA datasets.

### 2.3. Human Tissues

Human BC samples were kindly provided by patients who underwent BC surgery. All patients participating in the study gave informed consent, and protocols were approved by the institutional ethics committee.

### 2.4. Cell Culture

The immortalized breast cell line MCF-10A and three human BC cell lines (MCF-7, MDA-MB-231, and MDA-MB-468) were obtained from Harbin Medical University Cancer Hospital (Harbin, P.R. China). All cell lines were cultured at 37°C in a humidified atmosphere with 5% CO_2_. MCF-7 cells were cultured in MEM; MDA-MB-231 and MDA-MB-468 cells were cultured in L-15 medium supplemented with 10% fetal bovine serum (FBS), penicillin (100 U/ml), streptomycin (100 *μ*g/ml), and nonessential amino acids (1%); and MCF-10A cells were cultured in DMEM/F12 medium supplemented with 5% horse serum, 100 U/ml penicillin, 100 *μ*g/ml streptomycin, 0.5 *μ*g/ml hydrocortisone, 10 *μ*g/ml insulin, and 100 ng/ml cholera toxin. All reagents used for the experiments were from Sigma-Aldrich (Sigma, St. Louis, MO, USA); the media and serum were from Gibco, Thermo Fisher Scientific, Inc.

### 2.5. Plasmids and Transfection

To construct the TROAP knockdown lentiviral vector, the synthesized DNA sequence CCGGCGCCGTGGACCAGGAGAACCACTCGATGGTTCTCCTGGTCCACGGCGTTTTTG was inserted into the lentiviral vector GV115 linearized by Age1 and EcoR1. The full-length sequence of human TROAP was amplified by PCR using the TROAP expression plasmid pcDNA1-Tastin (Addgene, MA, USA) as a template, and the resulting sequence was inserted into the pLVX lentiviral vector (Addgene, MA, USA) for overexpression experiments. Lentivirus produced in HEK-293TN cells was used to infect MCF-7 human BC cells for 48 h. Stable knockdown and overexpression of TROAP were confirmed by RT-PCR and WB.

### 2.6. Cell Proliferation Assay

Prior to stimulating proliferation, cells at 70% confluence were starved in serum-free medium for 24 h and then treated with medium containing 10% FBS. Human MCF-7, MCF-7-siTROAP, and MCF-7-TROAP cells were seeded in 96-well plates at a density of 2000 cells per well for 24 h. Each plate contained three wells corresponding to each experimental condition (shTROAP and TROAP) and three control wells. At different times (24, 48, 72, and 96 hours), cells were incubated with 20 *μ*L of MTT (5 mg/mL, Sigma) for 4 h at 37°C in 5% CO_2_. The purple formazan crystals were dissolved in 100 *μ*l of dimethyl sulfoxide (DMSO, Sigma-Aldrich, Beijing, China), and the absorbance was monitored at 490 nm (iMark, Bio-Rad, Hercules, CA, USA).

### 2.7. Cell Invasion and Migration

We suspended 1 × 10^5^ MCF-7, MCF-7-siTROAP, and MCF-7-TROAP cells in 100 *μ*l of serum-free medium. The cells were added into the upper chambers of transwells (8 *μ*M, Corning, Tewksbury, MA, USA) in a 24-well plate with or without Matrigel (BD Biosciences, San Jose, CA); 700 *μ*l of complete medium containing 10% FBS was added to the bottom wells as the chemoattractant. After a 24 h incubation, the cells on the upper surface of the membrane were removed. The cells on the bottom membrane surface were fixed with 95% ethanol and stained with 0.1% crystal violet. Photographs were taken in three independent fields of each well under a light microscope at 10× (Lecia, Germany). All independent experiments were performed in triplicate.

### 2.8. RNA Extraction and Quantitative Real-Time (qRT-PCR) Assays

Total RNA was extracted from BC tissues and cells using TRIzol reagent (Invitrogen, Grand Island, NY, USA) following the manufacturer's instructions. Subsequently, 2 g of purified RNA from each sample was reverse-transcribed using an M-MLV Reverse Transcription System (Promega, Madison, WI, USA). TROAP expression levels were quantified using SYBR Master Mix (TAKARA, Dalian, China) and a Roche LightCycler® 96 instrument (Roche Molecular Biochemicals, Mannheim, Germany). The mRNA expression of glyceraldehyde-3-phosphate dehydrogenase (GAPDH) was used as an internal control. The PCR conditions were as follows: 95°C for 15 s, followed by 45 cycles of 95°C for 5 s and annealing and extension at 60°C for 30 s, and finishing with a melting curve analysis. The following primers were synthesized by Invitrogen Company (Shanghai, P.R. China): TROAP, 5′-CCTCCGGGGTGTATCTCCTAC-3′ (forward) and 5′-ACGGCGCACGATGTAACAG-3′ (reverse); GAPDH, 5′-TGACTTCAACAGCGACACCCA-3′ (forward) and 5′- CACCCTGTTGCTGTAGCCAAA-3′ (reverse). GAPDH mRNA was used as internal reference. The data were analyzed by the 2-∆∆Ct method. All experiments were performed in triplicate.

### 2.9. Western Blotting Assay

BC cells and tissues were harvested and lysed in lysis buffer (RIPA buffer, Beyotime Institute of Biotechnology, China) containing a protease inhibitor cocktail. Total cellular proteins were extracted and quantitated using the Bradford protein assay kit. Equivalent amounts of total protein were separated by 10% SDS polyacrylamide gel electrophoresis (SDS-PAGE) and then transferred to a polyvinylidene fluoride (PVDF) membrane, which was incubated overnight at 4°C with mouse monoclonal anti-TROAP antibody (1:2000) and mouse anti-human GAPDH (1:1000) antibodies diluted in TBS before incubation with the corresponding HRP-labeled secondary antibodies. The reaction was detected with ECL Plus detection reagent. GAPDH was used as an internal loading control. The protein bands were detected and visualized using Quantity One Software v4.62 (Bio-Rad, Hercules, CA, USA).

### 2.10. Cell Cycle Distribution

To observe the effect on the cell cycle distribution of MCF-7 and MCF-7-siTROAP cells by flow cytometry (FCM), cells were seeded at a density of 1 × 10^5^/ml in six-well plates for 24 h. Adherent cells were collected, washed with prechilled PBS, fixed in 2 mL of cold 75% ethanol overnight at 4°C, and resuspended in staining buffer (20 *μ*g/mL propidium iodide (PI; Sigma), 0.1% Triton X-100, and 0.2 mg/mL RNase in PBS) for 2 h at 4°C. The PI-stained cells were analyzed using a flow cytometer (Becton-Dickinson). Data analysis was performed using CXP Software (Beckman Coulter, Inc.). Each experiment was performed three times, and the ratios of cells in the G0/G1, S, and G2/M phases were determined and then expressed as the mean ± standard deviation (SD).

### 2.11. Statistical Analysis

The effects on cells in different groups were analyzed by one-way analysis of variance (ANOVA) using Sigma SPSS 13.0 (SPSS Inc., Chicago, IL, USA). Differences in TROAP expression in BC tissues and controls were evaluated by* t*-test. RNA-sequencing data were downloaded from the GEO database (https://www.ncbi.nlm.nih.gov/geo), which contained TROAP expression profiles in human BC. Gene correlation analysis was performed with the R2 platform (http://r2.amc.nl), and the genes and pathways associated with TROAP were analyzed by GO biological process and KEGG pathway analysis. The Kaplan-Meier method was used to evaluate survival data. mRNA expression data for BC and normal tissues were provided in the TCGA dataset. The differences in mRNA levels between BC and normal tissues were analyzed by one-way ANOVA. Data on the different molecular subtypes of BC were downloaded from the TCGA database. The differences in mRNA levels among tissues of different molecular subtypes of BC and normal tissues were evaluated by* t*-test. We extracted expression data on TROAP and correlated genes. All cell experiments were performed in triplicate, and the data are presented as the mean ± standard error. The correlations between TROAP and related genes were determined by Pearson's correlation test.* P*<0.05 was considered statistically significant.

## 3. Results

### 3.1. TROAP Is Upregulated in BC Tissues and Cell Lines

**Figure 1:**
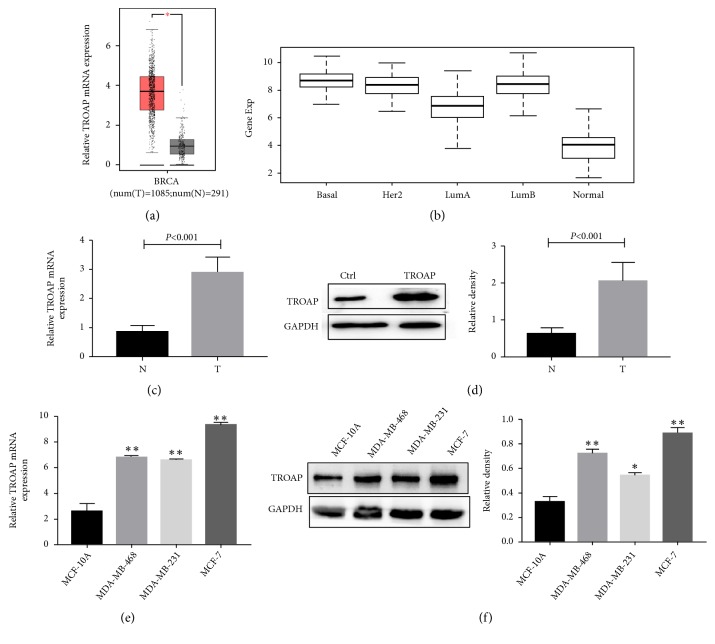
*TROAP is upregulated in BC tissues and cell lines*. (a) TROAP mRNA levels in 1085 BC tissues and 291 noncancerous tissues from the TCGA dataset. TROAP is upregulated in BC tissues (N, noncancerous; T, tumor;* P*<0.001). (b) TROAP mRNA levels in different molecular subtypes of BC and normal breast tissue. TROAP is upregulated in all molecular subtypes of BC (*P*<0.001). (c) TROAP mRNA levels are elevated in 30 pairs of BC tissues compared with matched tissues (*P*<0.001, n=30). (d) TROAP protein levels in 10 pairs of BC tissues and matched noncancerous tissues were detected by WB. (e) TROAP mRNA expression levels were determined by qRT-PCR in one immortalized breast cell line, MCF-10A, and three human BC cell lines, MCF-7, MDA-MB-231, and MDA-MB-468. (f) TROAP protein expression levels were determined by WB in one immortalized breast cell line, MCF-10A, and three human BC cell lines, MCF-7, MDA-MB-231, and MDA-MB-468 (*∗∗P*<0.001, *∗P*<0.005). The qRT-PCR and WB data are presented as the mean ± SD.

Our study analyzed the TCGA dataset and found that TROAP mRNA expression was significantly increased in BC tumor tissues (Figure 1(a); N, noncancerous; T, tumor;* P*<0.001) and in all molecular subtypes of BC (basal-like, HER2 overexpressed, luminal A and luminal B; Figure 1(b);* P*<0.001). We also examined TROAP expression status in BC tissues. TROAP mRNA was detected in 30 BC tissues and matched noncancerous adjacent tissues. The results demonstrated that TROAP mRNA expression was significantly higher in BC tumor tissues than in matched noncancerous adjacent tissues (Figure 1(c)).

We also detected TROAP protein expression levels in 10 pairs of tumor and noncancerous tissues and found them to be upregulated in all tumor tissues compared to matched noncancerous tissues (Figure 1(d)). To assess the role of TROAP in BC initiation and progression, TROAP expression was measured in one immortalized breast cell line, MCF-10A, and three human BC cell lines, MCF-7, MDA-MB-231, and MDA-MB-468. The noncancerous MCF-10A cells showed lower levels of both TROAP mRNA and protein compared to the tumor cell lines (Figures 1(e) and 1(f)). These results indicated that TROAP is increased in BC tissues and cell lines and may have a role in promoting BC development.

### 3.2. High Expression of TROAP Indicates a Poor Prognosis in BC

**Figure 2:**
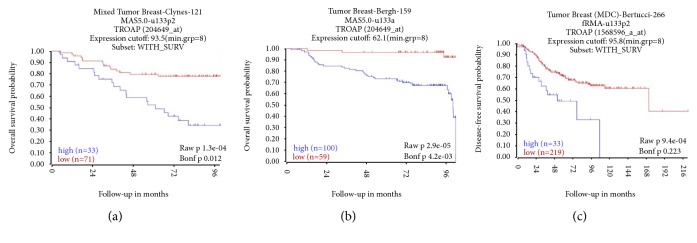
*The OS probability of cases with high TROAP expression was lower than that of those with low TROAP expression*. All cases were separated into two groups: high or low expression of TROAP. OS was then compared between the two groups using the Kaplan-Meier method. (a) OS from GEO dataset GSE42568 (104 BC patients; 14 samples lacked survival data and were omitted from the analysis, 33 cases had high TROAP levels, and 71 cases had low TROAP levels) (*P*<0.001). (b) OS from GEO dataset GSE1456 (159 BC patients; 100 cases with high TROAP expression and 59 cases with low TROAP expression) (*P*<0.001). (c) OS from the GEO dataset GSE21653 (266 BC patients; 14 samples lacked survival data and were omitted from the analysis, 33 cases had high TROAP levels, and 219 cases had low TROAP levels) (*P*<0.001).

We analyzed TROAP mRNA expression levels and clinical data for BC patients to estimate OS from three GEO datasets (GSE42568, 104 BC patients; GSE1456, 159 BC patients; GSE21653, 266 BC patients). Univariate Kaplan-Meier survival analysis revealed that OS was inversely associated with increased TROAP expression (Figure 2;* P*<0.001).

### 3.3. Function of TROAP and Its Related Genes Analyzed by KEGG and GO Analysis

**Figure 3:**
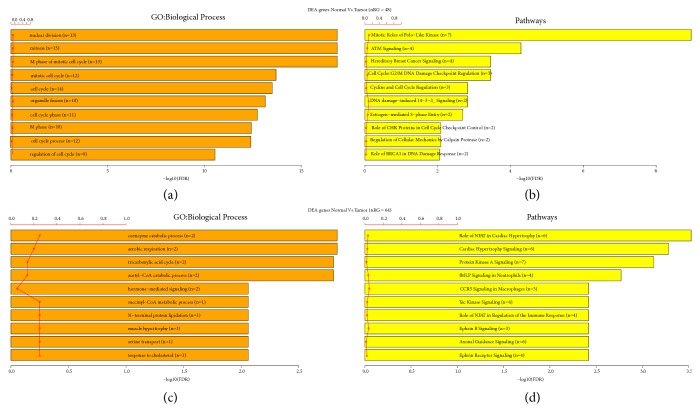
*GO and KEGG analyses*. (a) GO analysis: TROAP and positively correlated genes were associated with cell cycle and mitosis. (b) KEGG analysis: TROAP and positively correlated genes were associated with cell cycle and mitosis (*P*<0.001). (c) GO analysis: TROAP and negatively correlated genes were associated with cell proliferation (*P*<0.001). (d) KEGG analysis: TROAP and negatively correlated genes were associated with cell cycle control (*P*<0.001).

Overall, 4230 genes in the GSE42568 dataset were significantly correlated with TROAP expression (including 2357 positive and 1873 negative correlations), as were 2442 genes in the GSE1456 dataset (including 1261 positive and 1181 negative correlations) and 8708 genes in the GSE21653 dataset (including 5298 positive and 3410 negative correlations) by R2 platform analysis. The statistically significant positive and negative correlations at the intersection of the three datasets were selected, yielding 386 positively correlated genes (Supplemental Table 1) and 404 negatively correlated genes (Supplemental Table 2). To determine the function of TROAP and its related genes, we used GO analysis (Figure 2). Biological processes, including the cell cycle and mitosis, were associated with TROAP in BC (*P*<0.001, Figure 3(a)). KEGG analysis found that 107 genes were involved in ten signaling pathways (*P*<0.001, Figure 3(b)), most of which were related to the cell cycle and mitosis. Enrichment of negative_intersect_Gene revealed that biological processes such as regulation of cell proliferation were associated with TROAP in BC by GO analysis (Figure 3(c),* P*<0.001), and KEGG analysis confirmed that these genes were involved in cellular signaling pathways.

### 3.4. TROAP Affects BC Cell Proliferation and Growth

**Figure 4:**
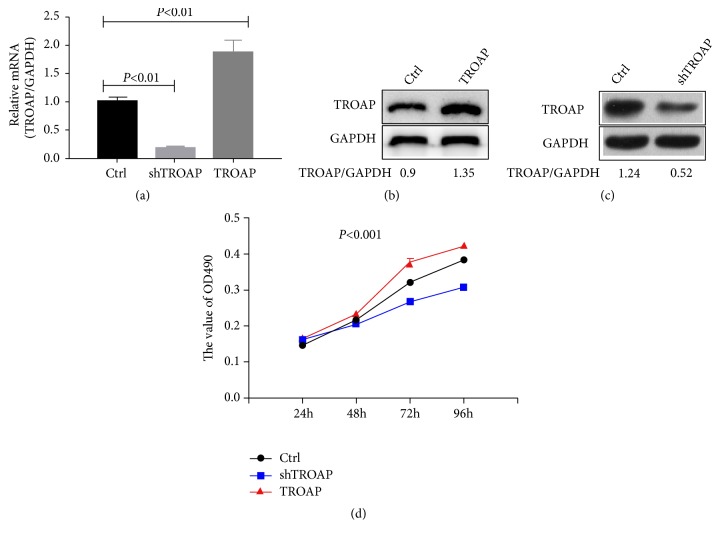
*The effects of TROAP depletion or plasmid-based overexpression*. (a) The transfection efficiency was verified by RT-PCR (*P*<0.01). (b) and (c) TROAP protein levels were higher in MCF-7 cells overexpressing TROAP than in control cells (*P*<0.001). (d) TROAP promoted MCF-7 cell proliferation. Cells were seeded in a 96-well plate at a density of 2 × 10^3^/well and analyzed after 24, 48, 72, or 96 hours. Cell viability and proliferation were determined by MTT assays. TROAP protein levels were lower in MCF-7 cells with TROAP depletion than in control cells (*P*<0.001).

To confirm the functional role of TROAP in BC development, we constructed TROAP knockdown and overexpression MCF-7 BC cell lines. TROAP mRNA and protein levels were verified in both the depleted and overexpressing cells (Figures 4(a)–4(c)). Then, we measured the effect of TROAP depletion or overexpression on BC cell growth. Cell proliferation was counting and measured using the MTT assay (Figure 4(d)). We found that TROAP enhanced MCF-7 cell proliferation. TROAP-overexpressing cells multiplied significantly faster than control cells. On the other hand, TROAP downregulation suppressed tumor cell proliferation (Figure 4(d)). Taken together, these results suggest that TROAP plays a protumorigenic role in BC cell growth.

### 3.5. TROAP Promotes BC Invasion and Migration of BC

**Figure 5:**
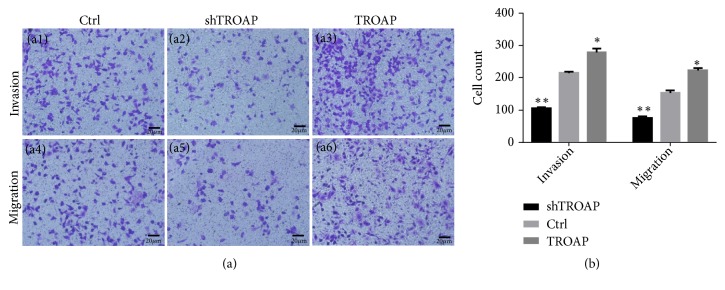
*TROAP depletion inhibits the migratory and invasive abilities of BC*. (a1)-(a3) The invasion of control, shTROAP, and TROAP-overexpressing MCF-7 cells (×400). (a4)-(a6) The migration of control, shTROAP, and TROAP-overexpressing MCF-7 cells s (×400). (b) TROAP depletion significantly inhibited the invasion and migration of MCF-7 cells (*P*< 0.05).

We evaluated the functional role of TROAP in BC migration and invasion using transwell assays. The invasion and migration of MCF-7 cells were markedly inhibited by TROAP depletion (*P*< 0.001) and significantly accelerated by TROAP overexpression (*P*< 0.05). These results demonstrated that TROAP facilitates the in vitro migration and invasion of BC (Figure 5).

### 3.6. TROAP Depletion Leads to G1/S Phase Arrest in BC Cells

**Figure 6:**
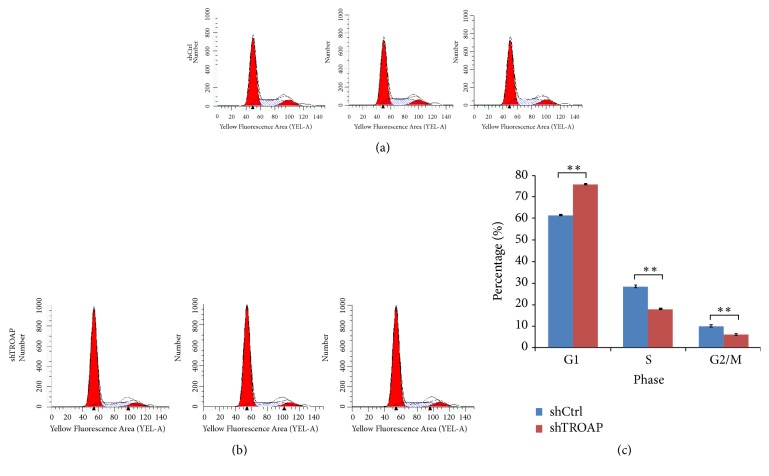
*TROAP depletion leads to G1/S phase arrest in BC cells*. FCM was performed to detect cell cycle distribution, and the percentages of cells in G0/G1, S, and G2/M phase were calculated. (a) The cell cycle distribution of control MCF-7 cells. (b) The cell cycle distribution of TROAP-depleted MCF-7 cells. (c) TROAP depletion leads to G1/S phase arrest in BC cells.

Cell cycle dysregulation has been shown to be involved in tumor growth inhibition. To clarify the underlying mechanism responsible for TROAP-mediated BC cell growth, we detected the cell cycle distribution of both TROAP-depleted and control cells by FCM. TROAP depletion in MCF-7 cells inhibited proliferation in vitro. In MCF-7-siTROAP BC cells, the proportion of cells in G1/S phase increased, and that in S and G2/M phase decreased, suggesting that TROAP could promote mitosis in BC cells (Figure 6).

## 4. Discussion

BC consists of a group of biologically and molecularly heterogeneous diseases that originate in the breast [3]. In China, BC has a markedly higher incidence and mortality than elsewhere in the world at 271.4 and 70.7 per 100,000 in 2015 [15], respectively. Therefore, there is an urgent need to identify more reliable diagnostic and prognostic biomarkers for the initiation, metastasis, and recurrence of BC.

TROAP was previously characterized as a requisite protein for cell adhesion that participates in early embryo implantation [16]. Many types of cancer express TROAP and human chorionic gonadotrophin (hCG), a marker of trophoblasts; for example, TROAP expression is observed in all cases of testicular germ cell tumors with lung metastasis [17]. Thus, trophoblasts and cancer cells may share a common mechanism that controls embryo implantation and the invasion and migration of malignant epithelial tumors. TROAP is also required for spindle assembly during mitosis [8, 18]. TROAP is localized on the centrosomes, microtubules, and mitotic spindle during the cell cycle. The major function of TROAP during mitosis is to maintain the structural and dynamic features of centrosomes, thereby contributing to spindle assembly [6]. High TROAP mRNA levels have been detected in the duodenum, esophagus, lymph node, placenta, skin, small intestine, stomach, bone marrow, and testis (https//www.ncbi.nlm.nih.gov/gene/10024). Consistent with these findings, high TROAP protein expression was also observed in various cancers compared to normal tissue, and this high expression promotes proliferation and migration and is involved in the poor prognosis of gallbladder cancer [9], ovarian cancer [10], colorectal cancer [11], liver cancer [12], lung cancer [19], and prostate cancer [20]. Moreover, TROAP may be associated with the invasion and migration of various cancers [21].

Our study analyzed 1085 BC tissues and 291 noncancerous tissues from the TCGA datasets and found that TROAP was upregulated in BC tissues. TROAP mRNA and protein levels were higher in BC tissue and three BC cell lines (MCF-7, MDA-MB-231, and MDA-MB-468) than in normal tissue and MCF-10A cells (from normal breast cells). This analysis revealed that TROAP was associated with BC. Furthermore, we analyzed TROAP mRNA expression levels and clinical follow-up survey data from BC patients to estimate OS from GEO datasets (GSE42568, GSE1456, and GSE21653). Univariate Kaplan-Meier survival analysis revealed that OS was inversely associated with high TROAP expression. To determine the function of TROAP and its related genes, highly positively and negatively correlated genes in the three datasets were analyzed, and the results confirmed that TROAP participates in the biological processes of cell cycle and mitosis in BC, as determined by GO and KEGG analyses. Subsequently, TROAP was knocked down and overexpressed in the current study to verify its functional role in MCF-7 cells. In vitro experiments demonstrated that TROAP depletion significantly suppressed cell proliferation, the G1 to S transition, and the migration and invasion of MCF-7 cells, whereas TROAP overexpression in BC cells had the opposite effects. Thus, TROAP plays a significant role in promoting tumor growth and metastasis and may be a potential diagnostic biomarker for BC.

In conclusion, the present study confirmed that TROAP contributes to BC cancer proliferation, invasion, and metastasis. TROAP may be a marker for predicting poor prognosis in BC.

## Data Availability

The data used to support the findings of this study are included within the article.

## Supplementary Materials

Supplementary Materials Supplemental Table 1: TROAP and its related positive genes in three GEO datasets. Supplemental Table 2: TROAP and its related negative genes in three GEO datasets.
