# Supplementary material for: TROAP Promotes Breast Cancer Proliferation and Metastasis
Source: Biomed Res Int. 2019 May 6;2019:6140951. doi: 10.1155/2019/6140951 (PMC6526557; doi:10.1155/2019/6140951)
Supplement: Supplementary Materials — Supplemental Table 1: TROAP and its related positive genes in three GEO datasets. Supplemental Table 2: TROAP and its related negative genes in three GEO datasets. [file 6140951.f1.pdf]

Supplemental table 1. TROAP and its related positive genes in three GEO dataset

Gene symbol	GSE42568		GSE21653		GSE1456	
	R-value	P-value	R-value	P value	R-value	P-value
ACAD10	0.417	1.48E-06	0.32	6.60E-07	0.314	0.0026285
ACTL6A	0.245	0.0085303	0.189	0.0046755	0.32	0.0021588
ALG3	0.356	6.27E-05	0.349	4.67E-08	0.338	0.0010768
AP1S1	0.257	0.0055639	0.34	1.06E-07	0.421	2.43E-05
APOBEC3B	0.429	6.29E-07	0.395	3.78E-10	0.447	6.38E-06
ARHGAP11A	0.283	0.0020076	0.521	3.98E-18	0.302	0.0041226
ARHGEF1	0.285	0.0018276	0.217	0.0010013	0.281	0.0081263
ARMC6	0.314	0.0005173	0.425	1.09E-11	0.408	4.73E-05
ARPC5L	0.254	0.0061395	0.424	1.16E-11	0.395	9.09E-05
ASF1B	0.748	4.96E-26	0.682	2.50E-35	0.632	1.05E-11
ASNS	0.299	0.0010191	0.354	2.84E-08	0.306	0.0035679
ASPM	0.551	9.99E-12	0.701	4.67E-38	0.59	6.08E-10
ATAD2	0.442	2.32E-07	0.461	7.28E-14	0.414	3.67E-05
ATAD3A	0.293	0.0013223	0.565	7.12E-22	0.485	6.95E-07
ATAD5	0.413	1.85E-06	0.39	6.52E-10	0.394	9.58E-05
ATAT1	0.28	0.0022399	0.38	1.87E-09	0.414	3.66E-05
ATP13A1	0.311	0.0005846	0.197	0.003088	0.529	4.65E-08
ATP2A2	0.324	0.0003163	0.396	3.32E-10	0.435	1.19E-05
AUNIP	0.47	2.54E-08	0.639	1.23E-29	0.488	5.82E-07
AURKA	0.65	3.01E-17	0.735	9.66E-44	0.625	2.45E-11
AURKB	0.519	3.57E-10	0.727	3.02E-42	0.413	3.78E-05
B4GALT2	0.287	0.00173	0.443	9.23E-13	0.3	0.0043254

BARD1	0.427	6.90E-07	0.349	4.48E-08	0.491	4.89E-07
BIRC5	0.548	1.38E-11	0.721	2.97E-41	0.535	3.40E-08
BLM	0.607	1.50E-14	0.765	3.54E-49	0.476	1.20E-06
BORA	0.425	8.27E-07	0.397	3.05E-10	0.418	2.98E-05
BRCA2	0.266	0.0039161	0.292	6.68E-06	0.429	1.64E-05
BRIP1	0.254	0.0063101	0.606	6.56E-26	0.478	1.11E-06
BRPF1	0.335	0.0001947	0.403	1.53E-10	0.299	0.0045133
BUB1	0.366	3.51E-05	0.655	1.25E-31	0.509	1.74E-07
BUB1B	0.564	2.64E-12	0.708	3.07E-39	0.53	4.57E-08
BYSL	0.438	3.18E-07	0.393	4.56E-10	0.31	0.0030781
C1orf106	0.271	0.0031809	0.423	1.38E-11	0.382	0.0001644
C1orf35	0.343	0.0001266	0.457	1.26E-13	0.447	6.27E-06
C6orf47	0.313	0.0005417	0.259	7.18E-05	0.368	0.0003134
CARM1	0.259	0.0050719	0.394	3.97E-10	0.354	0.0005661
CBS	0.28	0.0022909	0.389	7.19E-10	0.441	8.79E-06
CCHCR1	0.319	0.0004131	0.474	1.10E-14	0.462	2.70E-06
CCNA2	0.606	1.56E-14	0.627	3.43E-28	0.539	2.65E-08
CCNB1	0.576	6.53E-13	0.602	1.90E-25	0.451	5.08E-06
CCNB2	0.591	9.94E-14	0.72	4.20E-41	0.597	3.66E-10
CCNE1	0.403	3.82E-06	0.625	4.99E-28	0.421	2.46E-05
CCNE2	0.494	3.47E-09	0.491	7.13E-16	0.535	3.42E-08
CCNF	0.36	4.96E-05	0.687	4.92E-36	0.521	8.37E-08
CD3EAP	0.421	1.08E-06	0.429	5.94E-12	0.352	0.0006029
CDC20	0.591	1.00E-13	0.748	4.44E-46	0.557	7.69E-09
CDC25B	0.43	5.74E-07	0.527	1.50E-18	0.509	1.73E-07
CDC25C	0.49	4.81E-09	0.754	3.91E-47	0.491	4.90E-07

CDC6	0.568	1.65E-12	0.478	5.98E-15	0.382	0.0001645
CDC7	0.393	7.02E-06	0.53	7.48E-19	0.402	6.47E-05
CDCA3	0.485	7.24E-09	0.765	3.20E-49	0.636	9.20E-12
CDCA4	0.442	2.37E-07	0.584	1.08E-23	0.505	2.32E-07
CDCA8	0.559	4.47E-12	0.693	6.69E-37	0.497	3.49E-07
CDK1	0.621	2.03E-15	0.633	6.21E-29	0.542	2.23E-08
CDK2	0.404	3.51E-06	0.532	5.71E-19	0.295	0.005182
CDKN3	0.675	4.78E-19	0.655	1.25E-31	0.452	4.79E-06
CDT1	0.421	1.11E-06	0.731	6.09E-43	0.589	6.93E-10
CELSR3	0.291	0.0014052	0.326	3.84E-07	0.419	2.82E-05
CENPA	0.559	4.38E-12	0.642	4.57E-30	0.497	3.47E-07
CENPE	0.555	6.82E-12	0.703	2.47E-38	0.361	0.000424
CENPF	0.623	1.74E-15	0.742	7.09E-45	0.538	2.86E-08
CENPM	0.464	4.04E-08	0.663	1.04E-32	0.544	2.00E-08
CENPN	0.5	1.95E-09	0.611	1.83E-26	0.46	2.95E-06
CEP250	0.474	1.77E-08	0.55	1.51E-20	0.327	0.0016372
CEP55	0.578	5.22E-13	0.644	2.78E-30	0.542	2.20E-08
CFL1	0.435	3.92E-07	0.229	0.0005147	0.566	3.85E-09
CHAF1A	0.522	2.57E-10	0.692	8.45E-37	0.357	0.0005061
CHD7	0.246	0.008154	0.268	3.83E-05	0.404	5.88E-05
CHEK1	0.514	5.54E-10	0.546	3.53E-20	0.514	1.27E-07
CKAP5	0.329	0.000253	0.601	2.40E-25	0.303	0.0038945
CKS2	0.474	1.87E-08	0.581	2.21E-23	0.547	1.55E-08
CLCN2	0.319	0.0003985	0.373	4.13E-09	0.361	0.0004225
CORT	0.27	0.0033531	0.499	1.92E-16	0.403	5.99E-05
CPNE7	0.468	3.05E-08	0.244	0.0002019	0.384	0.0001456

CSK	0.33	0.0002434	0.387	9.32E-10	0.363	0.000377
CSTF2	0.375	2.13E-05	0.367	7.55E-09	0.415	3.45E-05
CTPS1	0.405	3.28E-06	0.503	9.38E-17	0.394	9.43E-05
CTRL	0.337	0.0001746	0.451	2.90E-13	0.39	0.0001138
DBF4	0.397	5.38E-06	0.439	1.70E-12	0.424	2.16E-05
DBNDD1	0.312	0.0005815	0.399	2.27E-10	0.38	0.0001825
DDX11	0.256	0.0057084	0.611	1.76E-26	0.536	3.17E-08
DDX39A	0.534	6.63E-11	0.636	2.64E-29	0.498	3.39E-07
DDX56	0.356	5.97E-05	0.39	6.33E-10	0.327	0.0016481
DEPDC1	0.45	1.26E-07	0.589	3.48E-24	0.599	3.41E-10
DHX34	0.247	0.0079263	0.394	4.12E-10	0.396	8.74E-05
DLGAP5	0.603	2.28E-14	0.652	2.81E-31	0.468	1.90E-06
DNA2	0.417	1.43E-06	0.528	1.08E-18	0.357	0.0004927
DNAJC9	0.459	6.19E-08	0.547	2.78E-20	0.477	1.19E-06
DNMT1	0.381	1.47E-05	0.426	9.44E-12	0.517	1.04E-07
DNMT3B	0.476	1.53E-08	0.543	6.65E-20	0.512	1.45E-07
DONSON	0.479	1.16E-08	0.534	4.03E-19	0.344	0.0008384
DPP3	0.289	0.0015501	0.238	0.0003015	0.419	2.85E-05
DSCC1	0.412	2.07E-06	0.627	3.47E-28	0.415	3.45E-05
DTL	0.587	1.67E-13	0.681	4.02E-35	0.603	2.69E-10
DTYMK	0.52	3.15E-10	0.566	6.41E-22	0.371	0.000264
DUS1L	0.272	0.0031308	0.396	3.18E-10	0.4	6.98E-05
E2F1	0.496	2.81E-09	0.729	1.13E-42	0.52	8.45E-08
E2F8	0.476	1.48E-08	0.627	3.17E-28	0.552	1.13E-08
ECT2	0.39	8.78E-06	0.395	3.69E-10	0.445	7.00E-06
EIF2AK1	0.325	0.0003011	0.281	1.48E-05	0.401	6.64E-05

EMD	0.362	4.29E-05	0.291	6.85E-06	0.355	0.0005386
EPHA1	0.267	0.00375	0.2	0.0025723	0.501	2.77E-07
ERCC6L	0.373	2.35E-05	0.584	1.31E-23	0.482	8.58E-07
ESPL1	0.583	2.98E-13	0.893	2.56E-89	0.667	3.19E-13
ESRP1	0.372	2.47E-05	0.285	1.10E-05	0.509	1.73E-07
ETV7	0.373	2.34E-05	0.404	1.40E-10	0.301	0.0042564
EXO1	0.572	1.03E-12	0.693	7.60E-37	0.485	7.10E-07
EXOSC4	0.302	0.0008882	0.304	2.62E-06	0.315	0.0025817
EZH2	0.507	1.07E-09	0.742	6.92E-45	0.602	2.77E-10
FA2H	0.283	0.0019903	0.196	0.0032083	0.351	0.0006359
FAM64A	0.546	1.80E-11	0.703	2.01E-38	0.456	3.89E-06
FANCC	0.302	0.0008945	0.5	1.54E-16	0.42	2.67E-05
FANCG	0.248	0.0076855	0.572	1.74E-22	0.647	2.57E-12
FANCI	0.503	1.57E-09	0.717	1.49E-40	0.503	2.58E-07
FARSA	0.335	0.0001942	0.332	2.23E-07	0.421	2.43E-05
FBRS	0.334	0.0001953	0.31	1.53E-06	0.328	0.0015863
FBXL6	0.402	4.04E-06	0.479	4.62E-15	0.577	1.67E-09
FBXO5	0.342	0.000134	0.391	5.72E-10	0.3	0.0043346
FEN1	0.403	3.82E-06	0.661	2.29E-32	0.425	2.06E-05
FER1L4	0.426	7.93E-07	0.371	4.99E-09	0.38	0.0001798
FKBP4	0.296	0.0011482	0.241	0.000237	0.461	2.86E-06
FOXM1	0.531	9.19E-11	0.791	9.38E-55	0.593	4.80E-10
GAPDH	0.276	0.0026872	0.361	1.42E-08	0.334	0.001264
GATAD2A	0.266	0.0040033	0.372	4.68E-09	0.34	0.0009745
GINS1	0.602	2.30E-14	0.694	4.84E-37	0.569	3.20E-09
GINS2	0.406	2.95E-06	0.552	1.06E-20	0.598	3.61E-10

GINS4	0.299	0.0010046	0.425	9.91E-12	0.388	0.0001232
GMEB2	0.299	0.0010067	0.384	1.32E-09	0.467	2.13E-06
GMNN	0.296	0.00116	0.374	3.67E-09	0.281	0.0082703
GMPPA	0.371	2.55E-05	0.185	0.005722	0.321	0.0020713
GMPS	0.318	0.00043	0.476	7.78E-15	0.385	0.0001448
GPR19	0.379	1.65E-05	0.567	4.94E-22	0.36	0.0004331
GPSM2	0.276	0.0027093	0.493	5.11E-16	0.332	0.0013317
GRK6	0.292	0.0013462	0.421	1.76E-11	0.379	0.000189
GRWD1	0.275	0.0027126	0.317	8.06E-07	0.401	6.81E-05
GTPBP3	0.406	3.12E-06	0.195	0.0033836	0.293	0.005415
H2AFX	0.581	3.74E-13	0.655	1.17E-31	0.457	3.66E-06
HAMP	0.328	0.0002718	0.3	3.37E-06	0.3	0.0044097
HAUS5	0.321	0.0003767	0.307	1.95E-06	0.511	1.49E-07
HAUS7	0.331	0.0002375	0.398	2.83E-10	0.279	0.0087598
HDGF	0.382	1.43E-05	0.555	5.78E-21	0.456	3.83E-06
HELLS	0.324	0.0003145	0.443	9.26E-13	0.584	1.04E-09
HGS	0.263	0.0045074	0.351	3.72E-08	0.445	6.98E-06
HIP1R	0.264	0.0042805	0.316	9.10E-07	0.481	9.11E-07
HJURP	0.6	2.96E-14	0.773	7.75E-51	0.497	3.57E-07
HMGA1	0.412	2.01E-06	0.409	7.59E-11	0.503	2.49E-07
HMGB2	0.476	1.55E-08	0.549	1.83E-20	0.35	0.000651
HMGB3	0.392	7.63E-06	0.514	1.50E-17	0.498	3.39E-07
HMGB3P1	0.39	8.32E-06	0.454	2.02E-13	0.393	0.0001006
HMMR	0.47	2.49E-08	0.589	4.16E-24	0.636	8.45E-12
HN1	0.48	1.11E-08	0.536	2.61E-19	0.353	0.000595
HNRNPAB	0.287	0.0017163	0.399	2.51E-10	0.279	0.0087528

HOXC11	0.265	0.0040575	0.244	0.0002059	0.458	3.34E-06
HPCAL1	0.273	0.0029498	0.351	3.75E-08	0.37	0.0002853
HSPBP1	0.333	0.0002047	0.346	6.29E-08	0.5	3.09E-07
IGHMBP2	0.344	0.0001209	0.353	3.05E-08	0.305	0.0036536
IKBKE	0.332	0.0002193	0.261	6.30E-05	0.376	0.0002172
IMPDH1	0.372	2.47E-05	0.421	1.61E-11	0.421	2.43E-05
IPO13	0.291	0.001412	0.368	6.58E-09	0.431	1.50E-05
IPO4	0.26	0.0048879	0.351	3.82E-08	0.527	5.69E-08
ISG15	0.242	0.0093914	0.238	0.0002938	0.324	0.0018638
JARID2	0.252	0.0066992	0.3	3.41E-06	0.562	5.45E-09
KCTD5	0.353	7.24E-05	0.233	0.0004003	0.32	0.0021414
KIAA0101	0.544	2.28E-11	0.613	1.31E-26	0.597	3.61E-10
KIF11	0.516	4.43E-10	0.605	9.21E-26	0.569	3.15E-09
KIF14	0.547	1.66E-11	0.69	1.96E-36	0.422	2.35E-05
KIF15	0.463	4.47E-08	0.723	1.07E-41	0.56	6.21E-09
KIF18A	0.44	2.74E-07	0.554	7.95E-21	0.357	0.0005016
KIF18B	0.557	5.59E-12	0.808	9.13E-59	0.689	2.15E-14
KIF20A	0.481	1.07E-08	0.76	2.37E-48	0.594	4.79E-10
KIF20B	0.257	0.005485	0.553	9.88E-21	0.303	0.0038656
KIF22	0.435	4.00E-07	0.518	7.26E-18	0.394	9.50E-05
KIF23	0.387	1.04E-05	0.626	4.09E-28	0.613	9.10E-11
KIF2C	0.639	1.39E-16	0.749	3.67E-46	0.611	1.04E-10
KIF4A	0.612	7.14E-15	0.742	7.31E-45	0.564	4.43E-09
KIFC1	0.621	2.05E-15	0.763	8.36E-49	0.618	5.01E-11
KLHDC4	0.335	0.0001912	0.427	7.74E-12	0.363	0.0003766
KPNA2	0.411	2.12E-06	0.61	2.71E-26	0.414	3.66E-05

LAD1	0.284	0.0019677	0.442	1.15E-12	0.401	6.85E-05
LAGE3	0.457	7.50E-08	0.358	1.84E-08	0.306	0.0035627
LHX2	0.28	0.0023027	0.385	1.16E-09	0.344	0.0008422
LIG1	0.472	2.27E-08	0.574	1.14E-22	0.453	4.56E-06
LLGL2	0.326	0.0002965	0.232	0.0004126	0.471	1.68E-06
LMNB1	0.503	1.56E-09	0.617	4.81E-27	0.517	1.03E-07
LMNB2	0.393	7.09E-06	0.532	5.32E-19	0.394	9.38E-05
LOC730101	0.36	5.00E-05	0.222	0.0007881	0.309	0.0032216
LRFN4	0.258	0.0054253	0.407	9.25E-11	0.316	0.0024821
LRP8	0.468	3.01E-08	0.452	2.73E-13	0.391	0.0001064
LRRC14	0.264	0.0043539	0.251	0.0001272	0.276	0.0096453
LSM4	0.405	3.24E-06	0.399	2.26E-10	0.315	0.0025744
LSR	0.269	0.0035414	0.294	5.41E-06	0.435	1.19E-05
LYPLA2	0.277	0.0025985	0.29	7.37E-06	0.423	2.26E-05
MAD2L1	0.536	5.55E-11	0.403	1.44E-10	0.368	0.0003035
MAP1S	0.319	0.0003998	0.271	3.19E-05	0.317	0.0023948
MARCKSL1	0.312	0.0005837	0.185	0.0055489	0.493	4.40E-07
MAZ	0.275	0.0028042	0.284	1.23E-05	0.502	2.76E-07
MC1R	0.32	0.0003918	0.37	5.69E-09	0.381	0.000174
MCM10	0.496	2.84E-09	0.628	2.55E-28	0.284	0.0074221
MCM2	0.573	1.02E-12	0.573	1.26E-22	0.519	9.15E-08
MCM3	0.367	3.24E-05	0.503	1.00E-16	0.364	0.0003669
MCM3AP-AS1	0.281	0.0021649	0.262	5.89E-05	0.357	0.0004926
MCM4	0.307	0.0007052	0.582	1.90E-23	0.53	4.53E-08
MCM5	0.482	9.73E-09	0.472	1.49E-14	0.343	0.0008676
MCM6	0.467	3.18E-08	0.516	1.09E-17	0.327	0.0016558

MCM7	0.341	0.0001404	0.572	1.77E-22	0.465	2.38E-06
MELK	0.548	1.40E-11	0.709	2.20E-39	0.461	2.88E-06
MEN1	0.304	0.0008127	0.22	0.000863	0.403	5.97E-05
METTL1	0.283	0.0019801	0.38	1.97E-09	0.465	2.35E-06
MIS18A	0.439	3.03E-07	0.48	4.19E-15	0.337	0.00112
MKI67	0.485	7.56E-09	0.734	1.63E-43	0.597	3.62E-10
MKRN3	0.282	0.0021394	0.232	0.0004358	0.306	0.0035738
MMP1	0.272	0.0031089	0.376	2.90E-09	0.395	8.96E-05
MRGBP	0.452	1.14E-07	0.59	2.91E-24	0.449	5.76E-06
MTHFD2	0.371	2.51E-05	0.306	2.21E-06	0.513	1.41E-07
MUTYH	0.347	0.0001043	0.536	2.62E-19	0.331	0.0014005
MYBL1	0.372	2.48E-05	0.395	3.73E-10	0.316	0.0024628
MYBL2	0.566	2.05E-12	0.775	3.37E-51	0.462	2.77E-06
MYO19	0.268	0.0036918	0.429	5.92E-12	0.56	6.23E-09
MYO9B	0.308	0.0006716	0.291	6.87E-06	0.318	0.0022595
NAT10	0.272	0.0031162	0.426	9.15E-12	0.459	3.26E-06
NCAPD2	0.38	1.53E-05	0.484	2.16E-15	0.364	0.000362
NCAPG	0.481	1.01E-08	0.754	3.75E-47	0.579	1.50E-09
NCAPG2	0.325	0.0003109	0.598	4.66E-25	0.486	6.40E-07
NCAPH	0.522	2.54E-10	0.725	6.22E-42	0.501	2.78E-07
NDC80	0.453	1.00E-07	0.663	1.18E-32	0.548	1.44E-08
NEIL3	0.486	6.63E-09	0.663	1.28E-32	0.648	2.49E-12
NEK2	0.592	9.63E-14	0.642	5.31E-30	0.519	9.12E-08
NEU3	0.294	0.0012886	0.185	0.0056313	0.374	0.0002391
NOP16	0.243	0.0092231	0.357	2.04E-08	0.398	7.96E-05
NOP2	0.36	4.96E-05	0.585	1.05E-23	0.496	3.78E-07

NPEPL1	0.317	0.0004425	0.24	0.0002646	0.306	0.0035972
NPM3	0.269	0.0034632	0.378	2.49E-09	0.279	0.0086516
NUP205	0.3	0.0009874	0.407	9.55E-11	0.302	0.0040223
NUP85	0.396	5.96E-06	0.483	2.86E-15	0.278	0.0089556
NUSAP1	0.647	4.20E-17	0.738	2.75E-44	0.636	7.92E-12
OIP5	0.615	4.36E-15	0.563	1.14E-21	0.432	1.37E-05
ORC6	0.421	1.12E-06	0.697	1.80E-37	0.566	3.87E-09
OTUB1	0.322	0.0003576	0.346	5.84E-08	0.493	4.35E-07
PA2G4	0.415	1.65E-06	0.525	2.09E-18	0.453	4.48E-06
PAFAH1B3	0.293	0.0013228	0.231	0.0004546	0.403	5.97E-05
PAICS	0.335	0.0001907	0.327	3.54E-07	0.44	9.20E-06
PARPBP	0.329	0.0002566	0.477	7.21E-15	0.372	0.0002609
PASK	0.378	1.72E-05	0.458	1.23E-13	0.516	1.12E-07
PBK	0.556	5.78E-12	0.59	2.96E-24	0.598	3.48E-10
PCNA	0.386	1.07E-05	0.439	1.61E-12	0.39	0.000112
PDCD11	0.242	0.0094209	0.367	7.30E-09	0.505	2.28E-07
PDIA4	0.256	0.0058602	0.175	0.0088668	0.384	0.0001481
PDSS1	0.428	6.65E-07	0.465	4.40E-14	0.42	2.60E-05
PFKL	0.268	0.0037009	0.29	7.63E-06	0.276	0.0095797
PITX1	0.243	0.0090162	0.229	0.0005134	0.393	0.0001001
PKMYT1	0.533	7.34E-11	0.773	7.01E-51	0.438	9.99E-06
PLOD3	0.279	0.0024072	0.371	5.29E-09	0.379	0.0001858
POLA2	0.293	0.0013229	0.592	1.93E-24	0.299	0.0045287
POLD1	0.372	2.49E-05	0.56	2.34E-21	0.388	0.0001267
POLE2	0.468	3.05E-08	0.471	1.59E-14	0.435	1.19E-05
POLQ	0.471	2.42E-08	0.762	8.45E-49	0.434	1.26E-05

POLR1C	0.357	5.76E-05	0.47	1.91E-14	0.535	3.47E-08
POLR2H	0.359	5.27E-05	0.346	5.94E-08	0.544	1.92E-08
POP1	0.296	0.0011489	0.389	7.17E-10	0.36	0.000441
PPAT	0.314	0.000516	0.347	5.61E-08	0.494	4.13E-07
PPFIA3	0.298	0.0010524	0.321	5.98E-07	0.398	7.93E-05
PPME1	0.291	0.0014358	0.341	9.91E-08	0.353	0.000583
PPP1R10	0.273	0.0030171	0.253	0.0001119	0.459	3.22E-06
PPP1R14B	0.389	9.05E-06	0.397	2.88E-10	0.332	0.0013752
PPP2R5D	0.303	0.0008661	0.415	3.39E-11	0.296	0.0050326
PPP6R1	0.289	0.0015355	0.315	9.94E-07	0.353	0.0005832
PRAME	0.459	6.32E-08	0.271	3.16E-05	0.277	0.0092106
PRC1	0.559	4.55E-12	0.763	6.33E-49	0.532	4.12E-08
PRKCSH	0.274	0.0029176	0.382	1.59E-09	0.433	1.33E-05
PSRC1	0.498	2.36E-09	0.612	1.57E-26	0.593	5.02E-10
PTBP1	0.316	0.0004612	0.426	8.53E-12	0.459	3.17E-06
PTMA	0.378	1.73E-05	0.29	7.34E-06	0.376	0.0002171
PTTG1	0.659	7.83E-18	0.756	1.73E-47	0.592	5.29E-10
PTTG3P	0.487	6.62E-09	0.709	2.99E-39	0.411	4.16E-05
PVR	0.449	1.36E-07	0.377	2.78E-09	0.283	0.0075617
PYCR1	0.39	8.36E-06	0.307	1.91E-06	0.478	1.09E-06
PYCRL	0.334	0.0001951	0.187	0.0051155	0.371	0.0002671
RABIF	0.401	4.22E-06	0.303	2.82E-06	0.364	0.0003723
RACGAP1	0.554	7.89E-12	0.638	1.66E-29	0.574	2.11E-09
RAD51	0.435	3.97E-07	0.652	3.01E-31	0.531	4.39E-08
RAD51AP1	0.479	1.16E-08	0.587	5.75E-24	0.452	4.64E-06
RAD54L	0.38	1.55E-05	0.796	6.71E-56	0.514	1.28E-07

RAE1	0.289	0.001536	0.435	2.69E-12	0.396	8.42E-05
RALY	0.494	3.42E-09	0.342	8.93E-08	0.455	4.06E-06
RAPGEFL1	0.292	0.0014042	0.273	2.79E-05	0.281	0.0080592
RASGRF1	0.4	4.38E-06	0.219	0.0009216	0.297	0.0048852
RBM14	0.281	0.0022181	0.423	1.37E-11	0.327	0.0016284
RBM38	0.47	2.61E-08	0.403	1.46E-10	0.374	0.0002387
RCC1	0.381	1.49E-05	0.343	7.89E-08	0.422	2.35E-05
RECQL4	0.404	3.40E-06	0.703	2.10E-38	0.554	9.79E-09
RECQL5	0.267	0.0038108	0.299	3.83E-06	0.279	0.0087761
RFC3	0.426	7.67E-07	0.306	2.20E-06	0.34	0.00099
RFC4	0.385	1.17E-05	0.559	2.59E-21	0.459	3.19E-06
RGS19	0.351	7.90E-05	0.286	1.01E-05	0.284	0.0073094
RHBDF2	0.293	0.0013313	0.369	6.16E-09	0.439	9.69E-06
RIBC2	0.292	0.001348	0.437	2.18E-12	0.471	1.63E-06
RMI1	0.476	1.57E-08	0.227	0.0005674	0.364	0.0003693
RNASEH2A	0.565	2.28E-12	0.563	1.10E-21	0.371	0.0002633
RPP38	0.381	1.48E-05	0.303	2.77E-06	0.293	0.0054137
RPS6KB2	0.373	2.33E-05	0.452	2.58E-13	0.432	1.43E-05
RRM2	0.586	1.82E-13	0.607	5.90E-26	0.542	2.25E-08
RUVBL2	0.293	0.0013004	0.424	1.15E-11	0.282	0.0078498
SAC3D1	0.47	2.56E-08	0.492	6.24E-16	0.435	1.20E-05
SCRIB	0.273	0.0029351	0.361	1.41E-08	0.471	1.63E-06
SDHAF1	0.325	0.0003115	0.205	0.0019635	0.287	0.0068011
SF3A2	0.305	0.0007956	0.314	1.13E-06	0.325	0.0017744
SF3B4	0.384	1.26E-05	0.402	1.76E-10	0.491	4.94E-07
SH2B2	0.293	0.0013319	0.291	7.26E-06	0.493	4.43E-07

SHCBP1	0.429	6.00E-07	0.51	2.74E-17	0.377	0.0002016
SHMT2	0.335	0.0001891	0.504	8.22E-17	0.355	0.0005478
SIRT7	0.299	0.00102	0.357	2.02E-08	0.364	0.0003724
SKIV2L	0.253	0.0064903	0.281	1.56E-05	0.348	0.000704
SLC12A9	0.277	0.0025295	0.279	1.75E-05	0.438	1.01E-05
SLC17A9	0.31	0.0006196	0.245	0.0001882	0.339	0.0010365
SLC29A2	0.304	0.0008066	0.277	2.05E-05	0.298	0.0045764
SLC2A1	0.394	6.74E-06	0.373	4.25E-09	0.423	2.31E-05
SLC35A2	0.362	4.28E-05	0.291	6.96E-06	0.427	1.78E-05
SLC38A7	0.373	2.35E-05	0.346	5.89E-08	0.39	0.0001141
SLC39A4	0.321	0.0003725	0.381	1.66E-09	0.497	3.46E-07
SLC52A2	0.509	8.80E-10	0.433	3.58E-12	0.434	1.25E-05
SLC7A5	0.426	7.63E-07	0.593	1.66E-24	0.523	7.21E-08
SMARCA4	0.245	0.0085744	0.363	1.12E-08	0.531	4.23E-08
SMC4	0.387	1.03E-05	0.381	1.82E-09	0.445	7.00E-06
SND1	0.353	7.26E-05	0.195	0.0033319	0.344	0.0008391
SNRPB	0.371	2.60E-05	0.276	2.27E-05	0.33	0.0014386
SNRPF	0.407	2.87E-06	0.419	2.29E-11	0.334	0.0012664
SPAG5	0.557	5.60E-12	0.702	3.25E-38	0.58	1.44E-09
SPC25	0.513	5.73E-10	0.526	1.63E-18	0.473	1.49E-06
SQLE	0.425	8.15E-07	0.244	0.0001942	0.458	3.30E-06
STIL	0.382	1.41E-05	0.595	1.05E-24	0.519	9.36E-08
STMN1	0.493	3.66E-09	0.577	6.37E-23	0.39	0.0001132
STX3	0.271	0.003262	0.217	0.0010309	0.418	2.88E-05
SUGP2	0.244	0.008901	0.196	0.0032123	0.521	8.28E-08
SUPT5H	0.353	7.28E-05	0.277	2.02E-05	0.362	0.0004013

SUV39H1	0.395	6.37E-06	0.644	2.91E-30	0.453	4.37E-06
TACC3	0.5	2.06E-09	0.791	1.06E-54	0.438	9.96E-06
TAF6	0.261	0.0047178	0.272	2.89E-05	0.342	0.0009046
TATDN2	0.284	0.0019226	0.284	1.23E-05	0.31	0.0030139
TCOF1	0.305	0.0007854	0.507	5.25E-17	0.311	0.0029771
TDO2	0.36	4.88E-05	0.197	0.0030742	0.33	0.0014357
TDP1	0.346	0.0001081	0.326	3.98E-07	0.371	0.0002667
TEX10	0.248	0.0077359	0.341	9.73E-08	0.42	2.58E-05
TGIF2	0.421	1.12E-06	0.345	6.80E-08	0.466	2.24E-06
THOP1	0.257	0.0054975	0.377	2.56E-09	0.464	2.43E-06
TIMELESS	0.627	9.96E-16	0.692	9.86E-37	0.485	7.21E-07
TK1	0.568	1.62E-12	0.618	3.45E-27	0.406	5.37E-05
TMC6	0.264	0.0043228	0.219	0.000906	0.285	0.0071968
TMEM132A	0.244	0.0086699	0.443	1.00E-12	0.524	7.06E-08
TMEM74B	0.258	0.0052912	0.22	0.0008715	0.335	0.0012204
TMEM97	0.456	7.86E-08	0.328	3.26E-07	0.492	4.79E-07
TMPO	0.485	7.57E-09	0.361	1.36E-08	0.322	0.0020023
TOMM34	0.368	3.05E-05	0.275	2.31E-05	0.494	4.13E-07
TOMM40	0.564	2.62E-12	0.534	4.07E-19	0.527	5.64E-08
TOP2A	0.506	1.12E-09	0.591	2.58E-24	0.572	2.56E-09
TPI1	0.361	4.60E-05	0.503	9.84E-17	0.36	0.0004409
TPX2	0.652	2.29E-17	0.711	1.41E-39	0.585	9.63E-10
TRAPPC10	0.309	0.0006603	0.189	0.0047024	0.407	4.98E-05
TRIM24	0.247	0.0079596	0.236	0.0003305	0.387	0.0001312
TRIM26	0.266	0.0039405	0.317	8.01E-07	0.38	0.0001795
TRIM28	0.283	0.0020019	0.239	0.000281	0.362	0.000409

TRIP13	0.603	2.16E-14	0.666	5.35E-33	0.482	8.51E-07
TROAP	1	0	1	0	1	0
TRPM2	0.329	0.0002579	0.294	5.41E-06	0.285	0.0072731
TSC22D4	0.32	0.000393	0.33	2.65E-07	0.389	0.0001199
TTK	0.489	5.48E-09	0.708	3.39E-39	0.51	1.66E-07
TTLL4	0.245	0.0084741	0.344	7.01E-08	0.33	0.0014674
TUBA1B	0.449	1.34E-07	0.427	7.46E-12	0.282	0.0079241
TUBA1C	0.407	2.78E-06	0.37	5.30E-09	0.286	0.0068128
TYMS	0.553	7.94E-12	0.602	1.98E-25	0.469	1.80E-06
U2AF2	0.25	0.0071707	0.371	5.14E-09	0.482	8.73E-07
UBE2C	0.69	2.94E-20	0.801	4.20E-57	0.656	9.58E-13
UBE2S	0.507	1.05E-09	0.574	1.13E-22	0.388	0.0001216
UBQLN4	0.338	0.0001629	0.532	5.78E-19	0.309	0.0031701
ULBP1	0.291	0.001418	0.205	0.0020047	0.323	0.0019324
VARS	0.317	0.0004408	0.391	5.69E-10	0.36	0.0004416
WDR4	0.337	0.0001744	0.344	7.26E-08	0.422	2.37E-05
WDR62	0.277	0.0025314	0.729	1.47E-42	0.408	4.85E-05
WDR76	0.358	5.60E-05	0.344	7.39E-08	0.289	0.0062411
WHSC1	0.326	0.0002978	0.54	1.16E-19	0.538	2.85E-08
XPO6	0.3	0.0009834	0.316	9.36E-07	0.432	1.42E-05
YIF1B	0.287	0.0017117	0.294	5.79E-06	0.325	0.0017547
ZMIZ2	0.246	0.0082259	0.295	5.00E-06	0.467	2.03E-06
ZMYM3	0.246	0.0083212	0.174	0.0093631	0.392	0.0001035
ZNF335	0.262	0.0047014	0.242	0.000227	0.456	3.80E-06
ZNF672	0.351	7.91E-05	0.266	4.55E-05	0.368	0.0003042
ZWINT	0.639	1.49E-16	0.681	3.99E-35	0.569	3.17E-09

Supplemental table 2. TROAP and its related negative genes in three GEO dataset

No.	Gene symbol	GSE21653		GSE1456		GSE42568	
		R-value	P-value	R-value	P value	R-value	P-value
	PTPRM	-0.34	1.06E-07	-0.445	7.01E-06	-0.399	4.82E-06
	ABCA6	-0.552	1.02E-20	-0.5	3.05E-07	-0.319	0.000412
	ABCA8	-0.532	5.71E-19	-0.487	6.11E-07	-0.323	0.000337
	ABLIM1	-0.185	0.005552	-0.291	0.00598	-0.357	5.82E-05
	ACVR1	-0.405	1.14E-10	-0.403	5.99E-05	-0.507	1.06E-09
	ADAMTS5	-0.311	1.44E-06	-0.466	2.21E-06	-0.312	0.000573
	ADD3	-0.302	3.02E-06	-0.359	0.000448	-0.311	0.000607
	ADRA2A	-0.511	2.29E-17	-0.428	1.76E-05	-0.392	7.35E-06
	AFF1	-0.368	6.77E-09	-0.398	7.81E-05	-0.513	6.09E-10
	AHNAK	-0.478	5.63E-15	-0.388	0.000126	-0.514	5.36E-10
	AKAP11	-0.457	1.25E-13	-0.495	3.93E-07	-0.356	6.07E-05
	AKAP12	-0.515	1.31E-17	-0.572	2.64E-09	-0.368	3.09E-05
	ALDH1A1	-0.47	1.89E-14	-0.495	4.12E-07	-0.327	0.000276
	ALDH1A2	-0.407	9.86E-11	-0.372	0.000258	-0.306	0.000764
	AMOTL2	-0.293	6.03E-06	-0.4	6.98E-05	-0.326	0.000296
	ANGPTL2	-0.31	1.56E-06	-0.491	4.80E-07	-0.393	6.85E-06
	ANK2	-0.456	1.50E-13	-0.487	6.23E-07	-0.34	0.000148
	ANKMY2	-0.324	4.36E-07	-0.408	4.72E-05	-0.346	0.000108
	ANXA6	-0.273	2.66E-05	-0.307	0.003426	-0.255	0.005964
	AOC3	-0.338	1.33E-07	-0.523	7.08E-08	-0.257	0.005483
	AOX1	-0.243	0.000214	-0.508	1.84E-07	-0.256	0.005796
	APC	-0.294	5.64E-06	-0.448	5.80E-06	-0.487	6.32E-09
	APPL2	-0.516	9.54E-18	-0.293	0.00551	-0.362	4.28E-05

AQP1	-0.376	2.80E-09	-0.374	0.000232	-0.249	0.007328
ARHGAP6	-0.444	7.75E-13	-0.282	0.007926	-0.356	6.17E-05
ARHGEF40	-0.349	4.45E-08	-0.365	0.000357	-0.284	0.001946
ARID5B	-0.35	4.16E-08	-0.384	0.000145	-0.336	0.000176
ARL6IP5	-0.35	4.20E-08	-0.365	0.000351	-0.483	9.03E-09
ARMCX1	-0.381	1.72E-09	-0.346	0.000773	-0.382	1.42E-05
ASPH	-0.215	0.001148	-0.366	0.00034	-0.352	7.60E-05
ATG14	-0.25	0.000133	-0.283	0.007736	-0.339	0.000154
ATRX	-0.29	7.59E-06	-0.296	0.004923	-0.321	0.000371
AXL	-0.396	3.44E-10	-0.359	0.000461	-0.389	9.19E-06
BCL2L2	-0.263	5.55E-05	-0.363	0.000386	-0.326	0.000295
BCL6	-0.191	0.00411	-0.319	0.002166	-0.309	0.000659
BDH2	-0.231	0.000461	-0.35	0.000662	-0.45	1.27E-07
BHLHE41	-0.441	1.22E-12	-0.283	0.00765	-0.284	0.001906
BHMT2	-0.399	2.27E-10	-0.478	1.10E-06	-0.34	0.000148
BICC1	-0.359	1.63E-08	-0.427	1.84E-05	-0.513	5.76E-10
BNIP3L	-0.425	1.02E-11	-0.529	4.71E-08	-0.302	0.000908
BTD	-0.372	4.54E-09	-0.375	0.00022	-0.275	0.002738
C1R	-0.326	3.96E-07	-0.31	0.003058	-0.464	4.11E-08
C1S	-0.295	5.22E-06	-0.319	0.00219	-0.4	4.35E-06
C3	-0.363	1.17E-08	-0.297	0.004757	-0.342	0.000134
C7	-0.476	8.40E-15	-0.32	0.00215	-0.258	0.005415
CALD1	-0.348	4.79E-08	-0.326	0.001731	-0.432	4.82E-07
CAST	-0.302	2.98E-06	-0.507	2.02E-07	-0.421	1.08E-06
CAT	-0.265	4.80E-05	-0.549	1.36E-08	-0.3	0.000979
CAV1	-0.451	3.23E-13	-0.541	2.40E-08	-0.496	2.68E-09

CAV2	-0.313	1.21E-06	-0.517	1.06E-07	-0.386	1.11E-05
CCND2	-0.267	4.21E-05	-0.321	0.002073	-0.367	3.28E-05
CD248	-0.267	4.26E-05	-0.373	0.000248	-0.357	5.75E-05
CD93	-0.403	1.49E-10	-0.293	0.005502	-0.373	2.35E-05
CD99	-0.185	0.005534	-0.317	0.002377	-0.326	0.000299
CDH5	-0.412	5.16E-11	-0.303	0.003884	-0.302	0.000904
CDO1	-0.375	3.37E-09	-0.544	1.95E-08	-0.273	0.002962
CFH	-0.417	2.80E-11	-0.483	7.85E-07	-0.474	1.83E-08
CHRDL1	-0.401	1.82E-10	-0.522	7.64E-08	-0.255	0.005965
CILP	-0.439	1.55E-12	-0.323	0.001931	-0.453	1.02E-07
CLDN5	-0.336	1.54E-07	-0.355	0.000536	-0.328	0.000273
CLIC2	-0.295	5.10E-06	-0.356	0.000524	-0.277	0.002514
CLN5	-0.349	4.46E-08	-0.357	0.000507	-0.293	0.001323
COL14A1	-0.547	2.84E-20	-0.329	0.001544	-0.454	9.03E-08
COL15A1	-0.348	5.02E-08	-0.301	0.004164	-0.297	0.00112
COL8A1	-0.282	1.36E-05	-0.284	0.007351	-0.277	0.002573
COLEC12	-0.363	1.11E-08	-0.28	0.008354	-0.379	1.68E-05
COPS4	-0.222	0.000777	-0.452	4.83E-06	-0.326	0.00029
COPZ2	-0.323	4.90E-07	-0.466	2.16E-06	-0.478	1.23E-08
CORO2B	-0.334	1.86E-07	-0.378	0.000198	-0.272	0.003151
COX7A1	-0.279	1.74E-05	-0.493	4.36E-07	-0.396	5.69E-06
CPA3	-0.45	3.63E-13	-0.359	0.000462	-0.364	3.86E-05
CPE	-0.497	2.56E-16	-0.44	9.27E-06	-0.288	0.001649
CREBL2	-0.474	1.12E-14	-0.509	1.73E-07	-0.328	0.000271
CRIM1	-0.472	1.55E-14	-0.384	0.000146	-0.342	0.000135
CRK	-0.27	3.38E-05	-0.478	1.06E-06	-0.289	0.001597

CSDE1	-0.259	7.14E-05	-0.462	2.66E-06	-0.281	0.002165
CSGALNACT1	-0.228	0.000552	-0.343	0.000867	-0.335	0.000192
CTSG	-0.309	1.60E-06	-0.309	0.003195	-0.284	0.001977
CTSO	-0.541	1.03E-19	-0.305	0.003671	-0.336	0.000181
CXCL12	-0.574	9.84E-23	-0.497	3.46E-07	-0.483	9.01E-09
CYBRD1	-0.665	5.48E-33	-0.492	4.64E-07	-0.416	1.57E-06
CYR61	-0.359	1.75E-08	-0.283	0.007588	-0.418	1.40E-06
DAAM2	-0.239	0.000277	-0.33	0.001448	-0.249	0.007467
DAB2	-0.374	3.49E-09	-0.515	1.23E-07	-0.275	0.002787
DCN	-0.552	9.89E-21	-0.43	1.52E-05	-0.489	5.15E-09
DDR2	-0.328	3.27E-07	-0.546	1.69E-08	-0.404	3.45E-06
DDX3X	-0.237	0.00032	-0.39	0.000113	-0.379	1.68E-05
DENND5B	-0.223	0.000739	-0.372	0.000252	-0.254	0.006138
DIXDC1	-0.642	4.66E-30	-0.5	2.98E-07	-0.478	1.34E-08
DLC1	-0.398	2.65E-10	-0.411	4.03E-05	-0.399	4.65E-06
DMXL1	-0.345	6.42E-08	-0.339	0.001024	-0.3	0.000984
DNAJB4	-0.224	0.000699	-0.488	5.89E-07	-0.285	0.001896
DOCK4	-0.36	1.56E-08	-0.286	0.007004	-0.258	0.00542
DPP4	-0.203	0.002231	-0.354	0.000573	-0.321	0.000371
DPT	-0.423	1.32E-11	-0.549	1.39E-08	-0.455	8.91E-08
DPYD	-0.439	1.62E-12	-0.43	1.51E-05	-0.442	2.37E-07
DPYSL2	-0.315	9.60E-07	-0.422	2.33E-05	-0.446	1.68E-07
DSE	-0.278	1.87E-05	-0.436	1.12E-05	-0.426	7.71E-07
DUSP1	-0.481	3.59E-15	-0.409	4.44E-05	-0.319	0.000411
DYNC1I2	-0.231	0.000455	-0.312	0.002846	-0.327	0.000285
EBF2	-0.303	2.69E-06	-0.418	2.93E-05	-0.245	0.008618

ECM2	-0.515	1.21E-17	-0.559	6.78E-09	-0.501	1.78E-09
EFCAB14	-0.478	6.07E-15	-0.406	5.27E-05	-0.438	3.19E-07
EFEMP1	-0.392	5.22E-10	-0.428	1.73E-05	-0.277	0.002564
EFEMP2	-0.248	0.000158	-0.28	0.008515	-0.443	2.25E-07
EGR1	-0.352	3.29E-08	-0.412	3.91E-05	-0.362	4.28E-05
EHD2	-0.305	2.34E-06	-0.351	0.00063	-0.555	6.38E-12
EID1	-0.445	7.21E-13	-0.445	7.05E-06	-0.336	0.000183
EIF1B	-0.309	1.65E-06	-0.406	5.37E-05	-0.455	8.55E-08
ELK3	-0.476	7.94E-15	-0.38	0.000184	-0.446	1.80E-07
EMCN	-0.536	2.38E-19	-0.314	0.002659	-0.302	0.000901
EML1	-0.283	1.31E-05	-0.326	0.001697	-0.414	1.77E-06
EMP1	-0.42	1.82E-11	-0.428	1.70E-05	-0.454	9.67E-08
EMX2	-0.185	0.005465	-0.348	0.000706	-0.451	1.17E-07
ENPP2	-0.303	2.68E-06	-0.427	1.83E-05	-0.323	0.000344
EOGT	-0.194	0.003516	-0.291	0.005879	-0.299	0.001004
EPAS1	-0.332	2.19E-07	-0.523	7.22E-08	-0.352	7.67E-05
EPS15	-0.271	3.19E-05	-0.374	0.000235	-0.296	0.00116
ETFDH	-0.278	1.90E-05	-0.503	2.58E-07	-0.27	0.003401
EVI5	-0.368	6.71E-09	-0.337	0.00111	-0.326	0.000289
EZH1	-0.278	1.94E-05	-0.345	0.000828	-0.483	8.50E-09
F13A1	-0.39	6.37E-10	-0.505	2.30E-07	-0.31	0.000637
F3	-0.356	2.19E-08	-0.443	7.72E-06	-0.301	0.000926
F8	-0.384	1.21E-09	-0.46	2.97E-06	-0.262	0.004703
FAM172A	-0.496	3.23E-16	-0.434	1.26E-05	-0.345	0.000112
FAM8A1	-0.218	0.000983	-0.333	0.001288	-0.367	3.38E-05
FAT4	-0.486	1.67E-15	-0.387	0.000129	-0.415	1.65E-06

FBLN1	-0.477	7.26E-15	-0.466	2.21E-06	-0.523	2.47E-10
FBLN2	-0.29	7.76E-06	-0.414	3.65E-05	-0.354	7.03E-05
FBLN5	-0.305	2.24E-06	-0.376	0.000211	-0.334	0.000202
FBN1	-0.403	1.49E-10	-0.439	9.95E-06	-0.48	1.13E-08
FBXL5	-0.555	6.18E-21	-0.533	4.08E-08	-0.438	3.21E-07
FBXO3	-0.414	4.20E-11	-0.406	5.31E-05	-0.347	0.0001
FCER1A	-0.406	1.10E-10	-0.404	5.88E-05	-0.286	0.001764
FERMT2	-0.297	4.26E-06	-0.539	2.64E-08	-0.444	1.96E-07
FGF1	-0.337	1.35E-07	-0.345	0.000805	-0.389	8.83E-06
FHL1	-0.473	1.25E-14	-0.55	1.30E-08	-0.387	1.02E-05
FILIP1L	-0.307	1.96E-06	-0.345	0.00082	-0.409	2.50E-06
FMOD	-0.512	2.20E-17	-0.453	4.38E-06	-0.312	0.000583
FNDC3A	-0.47	1.98E-14	-0.444	7.56E-06	-0.428	6.41E-07
FOS	-0.493	5.78E-16	-0.412	4.03E-05	-0.318	0.000419
FOSB	-0.381	1.72E-09	-0.293	0.005488	-0.277	0.002583
FOXO1	-0.474	1.03E-14	-0.408	4.84E-05	-0.357	5.85E-05
FSTL1	-0.356	2.24E-08	-0.416	3.29E-05	-0.493	3.75E-09
FXYD1	-0.469	2.34E-14	-0.393	9.87E-05	-0.268	0.003582
FYCO1	-0.444	8.41E-13	-0.332	0.001366	-0.503	1.58E-09
GABARAPL2	-0.196	0.003199	-0.352	0.000612	-0.265	0.004186
GAS1	-0.36	1.55E-08	-0.379	0.000186	-0.421	1.12E-06
GAS7	-0.391	5.67E-10	-0.371	0.000274	-0.35	8.43E-05
GCC2	-0.407	9.81E-11	-0.305	0.003605	-0.283	0.001998
GEM	-0.432	4.12E-12	-0.292	0.005708	-0.361	4.65E-05
GHR	-0.3	3.56E-06	-0.512	1.48E-07	-0.306	0.000764
GJA1	-0.442	1.05E-12	-0.286	0.006937	-0.304	0.000825

GLT8D2	-0.448	4.46E-13	-0.283	0.007662	-0.481	9.91E-09
GNAL	-0.246	0.000172	-0.445	7.10E-06	-0.256	0.005782
GNG11	-0.38	1.88E-09	-0.565	4.44E-09	-0.483	8.85E-09
GNG12	-0.558	3.25E-21	-0.353	0.000596	-0.515	5.16E-10
GOLIM4	-0.282	1.38E-05	-0.3	0.004335	-0.276	0.002664
GPRASP1	-0.422	1.57E-11	-0.34	0.000984	-0.272	0.003135
GPX3	-0.194	0.003491	-0.473	1.48E-06	-0.251	0.00685
GRK5	-0.221	0.000818	-0.376	0.000218	-0.26	0.004999
GSN	-0.34	1.08E-07	-0.535	3.52E-08	-0.375	2.04E-05
GULP1	-0.288	8.98E-06	-0.421	2.54E-05	-0.313	0.000542
HNMT	-0.524	2.58E-18	-0.509	1.76E-07	-0.468	3.09E-08
HNRNPA0	-0.353	2.95E-08	-0.283	0.00771	-0.367	3.36E-05
HOXA5	-0.333	2.07E-07	-0.299	0.004514	-0.252	0.006638
HOXA7	-0.265	4.69E-05	-0.389	0.000118	-0.346	0.000107
HSD17B11	-0.426	8.98E-12	-0.533	4.03E-08	-0.326	0.000289
HSD17B4	-0.193	0.003724	-0.331	0.001386	-0.379	1.66E-05
HTR2B	-0.411	6.02E-11	-0.356	0.00051	-0.264	0.004321
HTRA1	-0.461	7.81E-14	-0.387	0.000127	-0.507	1.07E-09
ID1	-0.335	1.73E-07	-0.308	0.003351	-0.301	0.000916
ID3	-0.248	0.000156	-0.289	0.006321	-0.359	5.07E-05
IGBP1	-0.341	9.95E-08	-0.29	0.006117	-0.261	0.00483
IGF1	-0.583	1.43E-23	-0.52	8.92E-08	-0.448	1.45E-07
IGFBP6	-0.447	5.77E-13	-0.553	1.04E-08	-0.422	1.06E-06
IL1R1	-0.395	3.84E-10	-0.315	0.002591	-0.379	1.67E-05
IL6ST	-0.575	8.07E-23	-0.277	0.009261	-0.318	0.000421
ILK	-0.217	0.001014	-0.287	0.006728	-0.321	0.000364

IRS2	-0.285	1.10E-05	-0.441	8.64E-06	-0.378	1.77E-05
ITGA7	-0.208	0.001651	-0.459	3.15E-06	-0.335	0.000193
ITGAV	-0.374	3.65E-09	-0.313	0.002722	-0.447	1.62E-07
ITIH5	-0.42	1.88E-11	-0.487	6.13E-07	-0.314	0.000524
ITM2A	-0.396	3.41E-10	-0.41	4.31E-05	-0.262	0.00467
ITSN1	-0.239	0.00027	-0.512	1.46E-07	-0.381	1.50E-05
JAK1	-0.194	0.003534	-0.424	2.08E-05	-0.518	3.74E-10
JAM2	-0.475	9.18E-15	-0.355	0.000548	-0.297	0.001094
JUN	-0.246	0.000173	-0.31	0.003059	-0.258	0.005371
KANK2	-0.362	1.30E-08	-0.431	1.49E-05	-0.432	5.01E-07
KCTD12	-0.524	2.30E-18	-0.506	2.11E-07	-0.432	4.82E-07
KIAA1109	-0.343	7.66E-08	-0.373	0.00024	-0.358	5.44E-05
KLF10	-0.26	6.78E-05	-0.343	0.000891	-0.34	0.000147
KLF2	-0.414	4.28E-11	-0.35	0.000662	-0.333	0.000212
KLF4	-0.318	7.59E-07	-0.522	7.77E-08	-0.272	0.003109
KLHL2	-0.296	4.80E-06	-0.379	0.000193	-0.316	0.00048
LAMA2	-0.575	9.08E-23	-0.443	7.97E-06	-0.419	1.27E-06
LAMA4	-0.275	2.29E-05	-0.499	3.12E-07	-0.391	7.92E-06
LAMC1	-0.233	0.000396	-0.4	6.96E-05	-0.446	1.80E-07
LAPTM4A	-0.283	1.26E-05	-0.337	0.001096	-0.338	0.000163
LDB2	-0.51	3.06E-17	-0.48	9.59E-07	-0.444	1.99E-07
LGALS3	-0.177	0.00825	-0.41	4.31E-05	-0.288	0.001667
LHFP	-0.549	1.86E-20	-0.469	1.89E-06	-0.433	4.41E-07
LIMA1	-0.498	2.44E-16	-0.422	2.41E-05	-0.398	5.19E-06
LIMCH1	-0.352	3.30E-08	-0.297	0.004881	-0.365	3.70E-05
LIPT1	-0.232	0.000432	-0.38	0.00018	-0.288	0.001666

LMBRD1	-0.375	3.18E-09	-0.454	4.20E-06	-0.384	1.26E-05
LMOD1	-0.416	3.31E-11	-0.322	0.001987	-0.483	8.55E-09
LOX	-0.289	8.31E-06	-0.285	0.007159	-0.447	1.62E-07
LPAR1	-0.487	1.44E-15	-0.364	0.000372	-0.481	9.90E-09
LPAR6	-0.441	1.25E-12	-0.359	0.000456	-0.41	2.35E-06
LPL	-0.305	2.30E-06	-0.471	1.66E-06	-0.25	0.007111
LPP	-0.331	2.39E-07	-0.348	0.000714	-0.261	0.004726
LRP1	-0.363	1.17E-08	-0.355	0.000529	-0.505	1.30E-09
LUM	-0.433	3.87E-12	-0.277	0.009387	-0.33	0.000242
MAF	-0.393	4.44E-10	-0.505	2.28E-07	-0.541	2.91E-11
MAFB	-0.385	1.10E-09	-0.289	0.00628	-0.267	0.003793
MAGEH1	-0.269	3.60E-05	-0.288	0.006538	-0.29	0.001518
MAN1A1	-0.417	2.78E-11	-0.476	1.22E-06	-0.415	1.65E-06
MAN1A2	-0.229	0.000522	-0.397	8.28E-05	-0.294	0.001275
MAP1B	-0.213	0.001258	-0.325	0.001743	-0.384	1.23E-05
MAP9	-0.328	3.34E-07	-0.277	0.009282	-0.25	0.007045
MAT2B	-0.256	9.26E-05	-0.333	0.001288	-0.295	0.001234
MBNL2	-0.26	7.10E-05	-0.409	4.54E-05	-0.376	2.04E-05
MCFD2	-0.266	4.67E-05	-0.445	6.83E-06	-0.273	0.003031
MDFIC	-0.226	0.000596	-0.532	4.15E-08	-0.291	0.001464
MED4	-0.338	1.23E-07	-0.292	0.005591	-0.267	0.00376
MEIS2	-0.387	9.49E-10	-0.365	0.000346	-0.32	0.000395
MEOX2	-0.487	1.40E-15	-0.461	2.86E-06	-0.282	0.002107
MFAP4	-0.597	5.26E-25	-0.431	1.47E-05	-0.494	3.34E-09
MITF	-0.343	8.17E-08	-0.448	5.83E-06	-0.273	0.00303
MMRN2	-0.465	3.88E-14	-0.362	0.000404	-0.247	0.007966

MN1	-0.453	2.49E-13	-0.298	0.004711	-0.413	1.87E-06
MOCS1	-0.293	6.14E-06	-0.275	0.009838	-0.259	0.005136
MORF4L1	-0.317	8.19E-07	-0.434	1.26E-05	-0.33	0.000245
MTMR6	-0.269	3.71E-05	-0.335	0.001209	-0.338	0.00016
MXRA7	-0.241	0.000238	-0.466	2.23E-06	-0.383	1.35E-05
MYCBP2	-0.188	0.004761	-0.36	0.000432	-0.3	0.000968
MYL9	-0.3	3.38E-06	-0.427	1.78E-05	-0.353	7.31E-05
N4BP2L1	-0.472	1.55E-14	-0.29	0.006031	-0.297	0.001115
NAALAD2	-0.409	7.11E-11	-0.371	0.000263	-0.244	0.008824
NAP1L3	-0.534	4.08E-19	-0.314	0.002647	-0.437	3.46E-07
NDN	-0.491	6.98E-16	-0.504	2.43E-07	-0.409	2.46E-06
NHLRC2	-0.273	2.74E-05	-0.378	0.000196	-0.261	0.004738
NID1	-0.309	1.72E-06	-0.337	0.001103	-0.499	2.17E-09
NOTCH2	-0.316	8.95E-07	-0.307	0.003443	-0.366	3.55E-05
NRP1	-0.425	1.07E-11	-0.295	0.005066	-0.352	7.61E-05
NSA2	-0.238	0.000295	-0.433	1.32E-05	-0.328	0.000264
NTRK2	-0.353	3.19E-08	-0.35	0.000669	-0.277	0.002586
NUAK1	-0.223	0.000746	-0.321	0.002056	-0.504	1.38E-09
OGN	-0.635	3.96E-29	-0.496	3.76E-07	-0.518	3.77E-10
OLFML1	-0.493	5.25E-16	-0.458	3.32E-06	-0.35	8.81E-05
OLFML3	-0.474	1.15E-14	-0.285	0.007205	-0.51	8.30E-10
OMD	-0.575	9.28E-23	-0.348	0.000714	-0.454	9.29E-08
OSBPL1A	-0.294	5.69E-06	-0.466	2.26E-06	-0.323	0.000337
PAFAH1B1	-0.401	1.86E-10	-0.404	5.70E-05	-0.469	2.67E-08
PALMD	-0.211	0.001409	-0.534	3.75E-08	-0.243	0.008951
PAMR1	-0.33	2.72E-07	-0.395	9.21E-05	-0.356	6.03E-05

PARVA	-0.417	2.79E-11	-0.395	8.80E-05	-0.374	2.20E-05
PCSK5	-0.357	1.98E-08	-0.434	1.26E-05	-0.438	3.20E-07
PCYOX1	-0.383	1.48E-09	-0.465	2.31E-06	-0.281	0.002156
PDE1A	-0.48	4.50E-15	-0.325	0.001775	-0.266	0.003891
PDGFC	-0.377	2.53E-09	-0.443	7.75E-06	-0.405	3.32E-06
PDGFD	-0.604	1.01E-25	-0.473	1.44E-06	-0.49	4.65E-09
PDGFRL	-0.48	3.95E-15	-0.474	1.41E-06	-0.48	1.14E-08
PDZD2	-0.209	0.001582	-0.477	1.19E-06	-0.38	1.60E-05
PDZD8	-0.287	9.53E-06	-0.435	1.20E-05	-0.449	1.43E-07
PDZRN3	-0.409	7.60E-11	-0.444	7.20E-06	-0.479	1.17E-08
PECAM1	-0.326	3.93E-07	-0.359	0.000462	-0.309	0.00067
PELI2	-0.47	1.81E-14	-0.34	0.000981	-0.344	0.000117
PEX11A	-0.231	0.000445	-0.392	0.000104	-0.344	0.000118
PGRMC2	-0.406	1.09E-10	-0.498	3.38E-07	-0.336	0.000176
PID1	-0.385	1.08E-09	-0.494	4.14E-07	-0.412	1.97E-06
PIK3C2A	-0.29	7.91E-06	-0.366	0.000338	-0.293	0.001343
PIK3R1	-0.448	5.03E-13	-0.4	7.02E-05	-0.288	0.00166
PIKFYVE	-0.225	0.000654	-0.333	0.00128	-0.27	0.003354
PJA1	-0.181	0.006947	-0.48	9.55E-07	-0.281	0.002221
PJA2	-0.573	1.37E-22	-0.436	1.13E-05	-0.376	1.97E-05
PKD2	-0.278	1.92E-05	-0.455	3.99E-06	-0.423	9.33E-07
PKIG	-0.259	7.50E-05	-0.31	0.003014	-0.316	0.000477
PLAGL1	-0.218	0.000946	-0.368	0.00031	-0.251	0.00685
PLCL2	-0.192	0.003867	-0.387	0.000132	-0.299	0.001005
PLN	-0.366	8.68E-09	-0.338	0.001072	-0.394	6.50E-06
PLS3	-0.211	0.001435	-0.39	0.000113	-0.417	1.48E-06

PLSCR4	-0.549	1.79E-20	-0.497	3.45E-07	-0.456	8.33E-08
PNMA1	-0.283	1.27E-05	-0.353	0.000587	-0.272	0.003166
PPP2CB	-0.336	1.61E-07	-0.382	0.000168	-0.388	9.49E-06
PPP2R5C	-0.412	5.03E-11	-0.354	0.000564	-0.325	0.000312
PPP3CB	-0.292	6.32E-06	-0.481	9.08E-07	-0.542	2.67E-11
PRELP	-0.213	0.001281	-0.44	8.99E-06	-0.287	0.001739
PRKD1	-0.249	0.000143	-0.381	0.000174	-0.265	0.00418
PRNP	-0.201	0.002439	-0.332	0.001357	-0.282	0.002147
PROS1	-0.464	4.88E-14	-0.512	1.46E-07	-0.432	4.82E-07
PTEN	-0.396	3.49E-10	-0.334	0.001248	-0.256	0.005784
PTGER3	-0.444	7.86E-13	-0.5	3.09E-07	-0.396	5.69E-06
PTGER4	-0.341	9.52E-08	-0.45	5.39E-06	-0.269	0.003529
PTGFR	-0.215	0.001169	-0.293	0.005473	-0.364	3.88E-05
PTPRG	-0.388	7.67E-10	-0.307	0.003415	-0.242	0.009299
PTRF	-0.366	8.67E-09	-0.471	1.66E-06	-0.499	2.18E-09
PURA	-0.455	1.69E-13	-0.282	0.007776	-0.4	4.60E-06
QDPR	-0.284	1.24E-05	-0.302	0.004126	-0.254	0.006167
RAPGEF2	-0.251	0.000124	-0.383	0.000153	-0.322	0.000359
RASA1	-0.3	3.51E-06	-0.312	0.002852	-0.356	6.19E-05
RBM7	-0.206	0.001903	-0.457	3.55E-06	-0.297	0.0011
RCAN2	-0.391	5.54E-10	-0.452	4.79E-06	-0.392	7.62E-06
RCBTB2	-0.356	2.17E-08	-0.413	3.71E-05	-0.408	2.72E-06
RECK	-0.519	5.67E-18	-0.52	8.60E-08	-0.564	2.56E-12
RERGL	-0.246	0.00018	-0.366	0.000335	-0.262	0.004702
RGCC	-0.282	1.42E-05	-0.477	1.16E-06	-0.279	0.002382
RHOBTB1	-0.175	0.008947	-0.283	0.00775	-0.27	0.003399

RNASE4	-0.584	1.29E-23	-0.514	1.29E-07	-0.437	3.44E-07
RNF11	-0.322	5.33E-07	-0.458	3.38E-06	-0.343	0.000123
ROCK1	-0.242	0.000231	-0.473	1.45E-06	-0.265	0.004174
RTN4	-0.216	0.001109	-0.356	0.00051	-0.25	0.007113
RUNX1T1	-0.53	7.80E-19	-0.428	1.70E-05	-0.376	2.04E-05
SACM1L	-0.278	1.85E-05	-0.428	1.76E-05	-0.334	0.000201
SAMD4A	-0.248	0.000159	-0.489	5.53E-07	-0.411	2.19E-06
SAR1A	-0.178	0.007885	-0.474	1.38E-06	-0.337	0.000171
SCP2	-0.364	1.07E-08	-0.433	1.32E-05	-0.346	0.000111
SEC23A	-0.267	4.22E-05	-0.499	3.29E-07	-0.437	3.35E-07
SEC24B	-0.345	6.35E-08	-0.444	7.41E-06	-0.261	0.004787
SEMA3C	-0.346	6.01E-08	-0.357	0.000488	-0.336	0.00018
SEPP1	-0.431	5.00E-12	-0.347	0.000758	-0.275	0.002759
SERINC1	-0.478	5.88E-15	-0.593	4.92E-10	-0.455	8.81E-08
SERPINF1	-0.382	1.55E-09	-0.441	8.79E-06	-0.374	2.22E-05
SERPING1	-0.304	2.43E-06	-0.352	0.000607	-0.384	1.23E-05
SFRP4	-0.471	1.58E-14	-0.329	0.001503	-0.382	1.42E-05
SGMS1	-0.291	6.93E-06	-0.386	0.000136	-0.288	0.001622
SH3BP5	-0.27	3.40E-05	-0.413	3.83E-05	-0.249	0.007462
SIRT1	-0.251	0.000127	-0.406	5.41E-05	-0.253	0.006525
SLC35G2	-0.224	0.000694	-0.377	0.000204	-0.247	0.007999
SLIT2	-0.491	6.83E-16	-0.361	0.000425	-0.31	0.000626
SLIT3	-0.437	2.26E-12	-0.337	0.001095	-0.321	0.000371
SLK	-0.397	3.01E-10	-0.305	0.003659	-0.327	0.000278
SNRK	-0.421	1.75E-11	-0.395	8.81E-05	-0.345	0.000113
SNX2	-0.325	4.30E-07	-0.509	1.73E-07	-0.278	0.002487

SOCS2	-0.383	1.44E-09	-0.317	0.002393	-0.312	0.000581
SOCS5	-0.288	8.61E-06	-0.298	0.004595	-0.244	0.008704
SOS2	-0.364	1.04E-08	-0.333	0.001324	-0.387	1.02E-05
SPARC	-0.404	1.42E-10	-0.318	0.002295	-0.372	2.49E-05
SPARCL1	-0.563	1.15E-21	-0.405	5.58E-05	-0.48	1.07E-08
SPG20	-0.36	1.53E-08	-0.457	3.58E-06	-0.518	3.69E-10
SPON1	-0.397	3.04E-10	-0.422	2.38E-05	-0.549	1.27E-11
SPRY1	-0.361	1.33E-08	-0.398	7.65E-05	-0.389	8.88E-06
SPRY2	-0.32	6.51E-07	-0.413	3.79E-05	-0.306	0.000742
SPTBN1	-0.233	0.000409	-0.512	1.47E-07	-0.248	0.007667
SPTLC1	-0.211	0.001464	-0.32	0.002093	-0.293	0.001316
SRPX	-0.388	8.53E-10	-0.488	5.69E-07	-0.418	1.40E-06
SSPN	-0.391	5.95E-10	-0.489	5.65E-07	-0.328	0.000266
STARD13	-0.518	7.24E-18	-0.284	0.007506	-0.384	1.23E-05
STAT5B	-0.322	5.29E-07	-0.43	1.51E-05	-0.297	0.001128
STEAP1	-0.261	6.33E-05	-0.471	1.68E-06	-0.244	0.008902
STOM	-0.422	1.55E-11	-0.289	0.006361	-0.416	1.54E-06
STRN3	-0.28	1.63E-05	-0.372	0.000253	-0.254	0.00629
STX12	-0.234	0.000385	-0.467	2.12E-06	-0.418	1.40E-06
SUCLA2	-0.313	1.15E-06	-0.563	5.00E-09	-0.304	0.000804
SVEP1	-0.51	2.87E-17	-0.499	3.23E-07	-0.444	2.10E-07
SYNC	-0.502	1.08E-16	-0.446	6.46E-06	-0.451	1.18E-07
SYNE1	-0.35	4.30E-08	-0.28	0.008426	-0.418	1.40E-06
SYNPO	-0.267	4.19E-05	-0.462	2.77E-06	-0.437	3.42E-07
TAB2	-0.228	0.000547	-0.276	0.009441	-0.271	0.003266
TACC1	-0.198	0.002839	-0.504	2.46E-07	-0.381	1.47E-05

TBC1D2B	-0.259	7.61E-05	-0.318	0.002318	-0.28	0.002292
TCF4	-0.485	1.99E-15	-0.33	0.001468	-0.45	1.34E-07
TCF7L2	-0.259	7.45E-05	-0.308	0.003276	-0.4	4.64E-06
TEK	-0.506	5.69E-17	-0.277	0.009331	-0.355	6.31E-05
TENM3	-0.3	3.55E-06	-0.421	2.50E-05	-0.269	0.003471
TGFBR2	-0.479	5.07E-15	-0.564	4.69E-09	-0.44	2.65E-07
TGFBR3	-0.376	2.80E-09	-0.39	0.000113	-0.298	0.00105
TGOLN2	-0.373	4.11E-09	-0.276	0.009533	-0.371	2.56E-05
THBD	-0.497	2.94E-16	-0.382	0.000168	-0.319	0.000409
TIMP3	-0.429	5.74E-12	-0.425	2.01E-05	-0.364	4.02E-05
TJP1	-0.357	2.09E-08	-0.367	0.000323	-0.498	2.40E-09
TLR3	-0.313	1.19E-06	-0.5	3.02E-07	-0.302	0.000894
TMEM47	-0.337	1.41E-07	-0.428	1.76E-05	-0.458	6.71E-08
TMEM59	-0.342	8.92E-08	-0.359	0.000453	-0.275	0.002785
TNS1	-0.341	9.84E-08	-0.444	7.32E-06	-0.375	2.09E-05
TNS3	-0.218	0.000965	-0.318	0.002264	-0.345	0.000112
TOR1AIP1	-0.381	1.69E-09	-0.304	0.003773	-0.334	0.000204
TRIM8	-0.441	1.22E-12	-0.343	0.000865	-0.4	4.51E-06
TSPAN31	-0.275	2.43E-05	-0.295	0.005098	-0.358	5.46E-05
TSPAN4	-0.251	0.000123	-0.336	0.001152	-0.281	0.002206
TSPYL1	-0.452	2.83E-13	-0.394	9.54E-05	-0.274	0.002924
TTC37	-0.466	3.60E-14	-0.47	1.75E-06	-0.444	2.11E-07
UBL3	-0.494	4.41E-16	-0.487	6.11E-07	-0.291	0.001412
UFSP2	-0.324	4.59E-07	-0.497	3.45E-07	-0.285	0.00188
UNC50	-0.216	0.001116	-0.319	0.002222	-0.291	0.001456
USP33	-0.318	7.66E-07	-0.424	2.16E-05	-0.267	0.003812

USP8	-0.288	8.67E-06	-0.503	2.49E-07	-0.302	0.000902
VGLL3	-0.183	0.006082	-0.501	2.93E-07	-0.345	0.000113
VPS13C	-0.376	3.02E-09	-0.32	0.002089	-0.406	2.93E-06
VWA8	-0.37	5.31E-09	-0.275	0.009983	-0.288	0.001638
WDR44	-0.307	2.03E-06	-0.388	0.000127	-0.241	0.009713
WISP2	-0.283	1.30E-05	-0.425	2.04E-05	-0.401	4.19E-06
WWTR1	-0.315	9.68E-07	-0.283	0.007642	-0.425	8.10E-07
XPA	-0.245	0.000184	-0.334	0.001262	-0.366	3.51E-05
YPEL5	-0.419	2.23E-11	-0.44	9.19E-06	-0.373	2.31E-05
ZBTB38	-0.43	5.39E-12	-0.449	5.78E-06	-0.244	0.008694
ZCCHC24	-0.576	7.63E-23	-0.505	2.29E-07	-0.413	1.91E-06
ZDHHC17	-0.306	2.19E-06	-0.344	0.000838	-0.285	0.001882
ZEB2	-0.46	8.18E-14	-0.513	1.34E-07	-0.346	0.000105
ZFP36L2	-0.372	4.59E-09	-0.338	0.00106	-0.433	4.49E-07
ZFPM2	-0.39	6.38E-10	-0.411	4.19E-05	-0.401	4.22E-06
ZNF189	-0.257	8.41E-05	-0.34	0.001004	-0.28	0.002261
ZNF423	-0.43	5.66E-12	-0.368	0.000302	-0.384	1.26E-05
